# Biologicals and small molecules in psoriasis: A systematic review of economic evaluations

**DOI:** 10.1371/journal.pone.0189765

**Published:** 2018-01-03

**Authors:** Christian Kromer, Daniel Celis, Diana Sonntag, Wiebke K. Peitsch

**Affiliations:** 1 Department of Dermatology, University Medical Center Göttingen, Göttingen University, Göttingen, Germany; 2 Mannheim Institute of Public Health, Social and Preventive Medicine, Medical Faculty Mannheim, Heidelberg University, Mannheim, Germany; 3 Faculty of Economic Sciences, Göttingen University, Göttingen, Germany; 4 Department of Health Science, University of York, Heslington, York, United Kingdom; 5 Department of Dermatology and Phlebology, Vivantes Klinikum im Friedrichshain, Berlin, Germany; Kinki Daigaku, JAPAN

## Abstract

Biological therapy for moderate-to-severe psoriasis is highly effective but cost-intensive. This systematic review aimed at analyzing evidence on the cost-effectiveness of biological treatment of moderate-to-severe psoriasis. A literature search was conducted until 30/06/2017 in PubMed, Cochrane Library, LILACS, and EconLit. The quality of identified studies was assessed with the checklist by the Centre for Reviews and Dissemination guidance. Out of 482 records, 53 publications were eligible for inclusion. Half of the studies met between 20 and 25 of the quality checklist items, displaying moderate quality. Due to heterogeneity of studies, a qualitative synthesis was conducted. Cost ranges per outcome were enormous across different studies due to diversity in assumptions and model design. Pairwise comparisons of biologicals revealed conflicting results. Overall, adalimumab appeared to be most cost-effective (100% of all aggregated pairwise comparisons), followed by ustekinumab (66.7%), and infliximab (60%). However, in study conclusions most recent publications favored secukinumab and apremilast (75% and 60% of the studies investigating these medications). Accepted willingness-to-pay thresholds varied between 30,000 and 50,000 USD/Quality-Adjusted Life Year (QALY). Three-quarters of studies were financially supported, and in 90% of those, results were consistent with the funder’s interest. Economic evaluation of biologicals is crucial for responsible allocation of health care resources. In addition to summarizing the actual evidence this review highlights gaps and needs for future research.

## Introduction

Psoriasis is a chronic inflammatory disease of the skin and joints with a prevalence of 1–3% world-wide [1], varying between different ethnicities and geographical regions [2, 3]. Patients often suffer from social and professional stigmatization as well as from cardiovascular, metabolic and psychiatric comorbidities [4]. Therefore, psoriasis can lead to an enormous reduction of health-related quality of life [5] as well as to considerable impairment of work productivity [6]. As psoriasis is incurable and mostly takes an either chronic-persistent or a frequently relapsing course, lifelong disease control is necessary. Therapeutic options comprise topical treatment, phototherapy, traditional systemic medication, and biologicals. First introduced in 2003, biologicals are highly effective in treatment of moderate-to-severe psoriasis but also cost-intensive [7].

The economic burden of psoriasis is known to be significant and has increased with the introduction of biologicals due to high medication costs. A Canadian study estimated the mean annual cost per patient to be as high as 6,278 USD with 57% direct cost (i.e. expenditure on medication, physician visits, laboratory testing etc.) and 43% indirect cost (i.e. loss of productivity due to absenteeism from work) [8]. A systematic review from the United States with a societal perspective found an annual expenditure of 15,135–18,243 USD per patient [9]. A recent Swedish study showed increased direct cost (+1,365 USD) and indirect costs (+ 3,319 USD) per patient with psoriasis per year, compared with the general population. If treated with biologicals, the high direct cost of medication (+ 23,293 USD per patient per year) was only partially offset by savings in indirect costs [10].

Several meta-analyses have shown an increased risk of cardiovascular events in patients with psoriasis [11, 12]. Samarasekera and colleagues reported hazard ratios of 3.04 for myocardial infarction, 1.59 for stroke, and 1.37 for cardiovascular mortality in case of severe psoriasis [12]. TNF-inhibitors were demonstrated to reduce the risk of cardiovascular events in psoriasis [13, 14]. As a consequence, biological treatment could be beneficial from a societal and economic viewpoint by reducing cardiovascular morbidity and mortality and thus increasing QALYs as well as by diminishing expenditure for management of cardiovascular comorbidity and associated events.

In order to efficiently allocate constrained resources in the health care sector, economic evaluation (e.g., cost-effectiveness analysis or cost-utility analysis) is crucial. Several systematic reviews have been carried out to compare the cost-effectiveness of psoriasis treatments in general [15–17], as well as biological therapies in particular [18, 19]. However, they found conflicting empirical evidence. The studies included were heterogeneous, and synthesis was either performed by quoting the study results [15, 18, 19], reporting cost-effectiveness ranges with wide intervals of incremental cost-effectiveness ratios (ICERs) [17], or focusing on the quality of included publications [16]. Thus, meaningful conclusions on the relative cost-effectiveness of biological agents could not be drawn. In the most recent review [17], abstracts and posters were excluded, potentially leading to an incomplete capture of economic data. Furthermore, three newly approved therapies, the interleukin 17A antibodies secukinumab and ixekizumab and a small molecule inhibiting phosphodiesterase 4, apremilast, were not considered. To date, secukinumab and ixekizumab are considered the most effective biologicals while apremilast provides a favorable risk profile.

This systematic review aims at collecting and synthesizing the available evidence on economic evaluations of biologicals for treatment of moderate-to-severe psoriasis, including newly approved biologicals and the small molecule apremilast. The quality of the included studies was critically evaluated.

## Methods

The design of the systematic review followed the Preferred Reporting Items for Systematic Reviews and Meta-Analysis (PRISMA) guidance (S1 Table) [20].

### Literature search

A literature search was conducted by CK in the electronic databases PubMed, Cochrane Library, LILACS, and EconLit from their inceptions until 30/06/2017. Search terms are shown in S1 Fig, for detailed search strings see S2 Table. Hand searches retrieved from the reference list of published reviews complemented these records.

### Study identification

After removal of duplicates, records were screened according to the following pre-specified inclusion criteria:

1. **Disease:** Moderate-to-severe psoriasis.
2. **Intervention and comparator:** Treatment with one of the currently or formerly approved biologicals or small molecules for psoriasis compared with any other treatment option or placebo.
3. **Outcome:** Disease- or patient-related health outcomes, i.e., reduction of the Psoriasis Area and Severity Index (PASI) by 50, 75 or 90% (PASI 50, 75 or 90), Physician’s Global Assessment (PGA), Dermatology Life Quality Index (DLQI), or Quality-Adjusted Life Years (QALYs).
4. **Economic evaluation:** Reporting of costs, outcomes and cost-effectiveness measures, e.g., ICERs.
5. **Type of publication:** Peer-reviewed journal articles, abstracts and posters.
6. **Language:** English, German, or Spanish language because most analyses were published in these languages in the last years.

**Fig 1:**
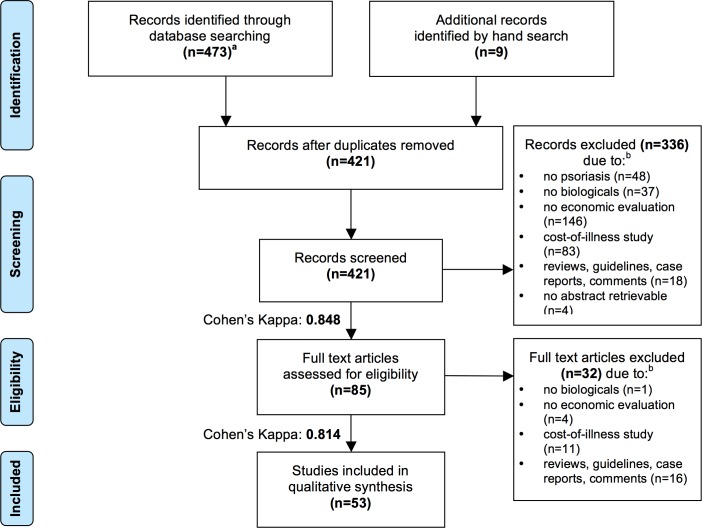
PRISMA Flow Diagram. ^a^ PubMed (n = 395), Cochrane (n = 68), LILACS (n = 6), EconLit (n = 4); period of search: from databases’ inceptions until 30/06/2017. ^b^ If more than one exclusion criterion applied, the record was assigned to the first applicable category in the order shown in the figure.

Exclusion criteria are listed in Fig 1.

The screening for eligibility was independently performed by two reviewers (CK and DC) at two levels ((i) titles and abstracts and (ii) full-text articles). Discrepancies were discussed until a consensus was reached. To measure inter-rater reliability, Cohen’s kappa was calculated in both screening stages [21].

### Quality assessment

The quality of eligible studies was assessed by CK with the checklist proposed by the Centre for Reviews and Dissemination guidance for undertaking systematic reviews [22]. This qualitative instrument consists of 36 items related to study design, data collection, and analysis and interpretation of results that can be answered with “yes”, “no”, or “not applicable” (for details on the items, see Supporting Information, S3 Table). It is based on Drummond’s expanded BMJ checklist [23], which remains one of the most commonly used instruments despite existence of other quality assessment tools [24–26]. The applied qualitative checklist precludes calculation of a quantitative score, as there are no defined values assigned to each checklist item. Thus, comparing the quality of included studies quantitatively or applying a weight to study results according to the quality was not performed in this review.

### Data extraction and analysis

Data from eligible studies were extracted by CK with respect to country, perspective, study type, model design, time horizon, discounting, comparators, effectiveness measure, utility valuation, included costs, results, sensitivity analysis (type, varied parameters, and conclusions), study conclusions, and funding information (funder, funder’s medical product for psoriasis, and consistency). To enable a comparison between results, costs were converted to 2015 USD using country-specific inflators in health care and purchasing power parity.

Due to heterogeneous designs, a qualitative synthesis was conducted. Cost ranges per PASI 75 response, DLQI minimal important difference (DLQI MID, i.e., a reduction by 5 points [27]), and QALY were summarized for all biologicals. Since broad intervals with overlap between the biologicals resulted, study findings were stratified according to the time horizon.

Moreover, pairwise comparisons were performed. The economic relationship between two comparators in each study was categorized into “dominant”, “dominated”, “higher benefit at higher cost”, and “lower benefit at lower cost” for studies that directly calculated ICERs between biologicals. If sensitivity analysis was performed, only the ICERs of the baseline scenario were included. Many studies investigated cost per outcome for biologicals compared to non-biological therapies. Results of these studies could not be incorporated into the matrix described above, which only displays direct biological-to-biological economic relationship. Instead, reported ICERs of biological vs. non-biological therapy were compared between the different biologicals considered in the particular study. The results of these “indirect” pairwise comparisons were categorized into “more cost-effective” (lower cost per benefit), “equally cost-effective” (similar cost per benefit), and “less cost-effective” (higher cost per benefit). For example, if one study compared biological A and biological B both to non-biological therapy and the resulting ICER for biological A was lower than the respective ICER for biological B, biological A was grouped into the category “more cost-effective”, as it can be assumed that treatment with biological A costs less than treatment with biological B to reach the same effect (or that biological A provides higher effectiveness at same costs). The number of studies in one category divided by the number of all studies investigating one pairwise comparison was calculated and the category with the highest share was assumed to display the most accurate economic relationship between two biologicals.

Moreover, the numbers of studies in which the preferred biological was stated by the authors in the conclusions were grouped according to the different biologicals and presented as percentage of all studies incorporating this biological. Finally, funding information according to the categories “not funded”, “funded but not consistent with funder’s interest”, and “funded and consistent with funder’s interest” was analyzed for all studies and segregated for individual biologicals.

## Results

### Literature search

Overall, 482 records were identified. 53 studies [28–80] were considered eligible for qualitative analysis according to inclusion and exclusion criteria (Fig 1). Cohen’s kappa was 0.848 for title and abstract screening and 0.814 for full-text screening, reflecting high inter-rater reliability.

### Quality assessment

For 15 of the 53 studies included, only an abstract or a poster could be retrieved, leading to lower quality data due to limited information [31, 33, 35, 38, 39, 43–47, 54, 57, 62, 77, 80].

All studies provided a research question; however, the viewpoint of the analyses was stated clearly in only 77% [28, 29, 32–35, 37–39, 41–52, 54–57, 59, 61, 62, 64–66, 70–76, 78–80]. A total of 47 studies reported the source of effectiveness estimates [28–42, 48–79], and in nearly two-thirds of them, further information on methods of synthesis (if based on multiple studies) or design and result (if based on a single study) was provided [28, 30, 31, 33–36, 38, 40, 48, 52, 54, 55, 58–60, 63–66, 69–74, 78, 79]. Even though price data was always recorded, only 31 studies reported quantities of resources and unit costs separately [28, 29, 32, 34, 35, 38, 40–42, 48–50, 52–55, 59–62, 64–67, 70–73, 75, 76, 78]. Nearly all studies stated the time horizon of analysis [28, 30, 31, 33–52, 54–72, 74–80]. 43% did not report statistical tests or confidence intervals [29, 32, 35, 37–41, 43–47, 49–51, 57, 61, 63, 66, 67, 75, 80]. Sensitivity analysis was clearly recorded in 42 studies [28, 30, 32–37, 39, 41–49, 51, 52, 54–57, 59–62, 64–66, 68–70, 72–74, 76–80]. Conclusions were drawn in all studies and three-quarters reported limitations [28, 30, 34–37, 40–42, 48–52, 54–57, 59–67, 69–76, 78, 79].

More than half of all studies met between 20 and 25 checklist items [28–30, 32–37, 39, 41, 42, 48, 49, 51–54, 57, 60, 62, 64, 66, 69, 71, 72, 74, 76, 77, 79], reflecting an overall moderate quality (S3 and S4 Tables).

### Qualitative synthesis

**Fig 2:**
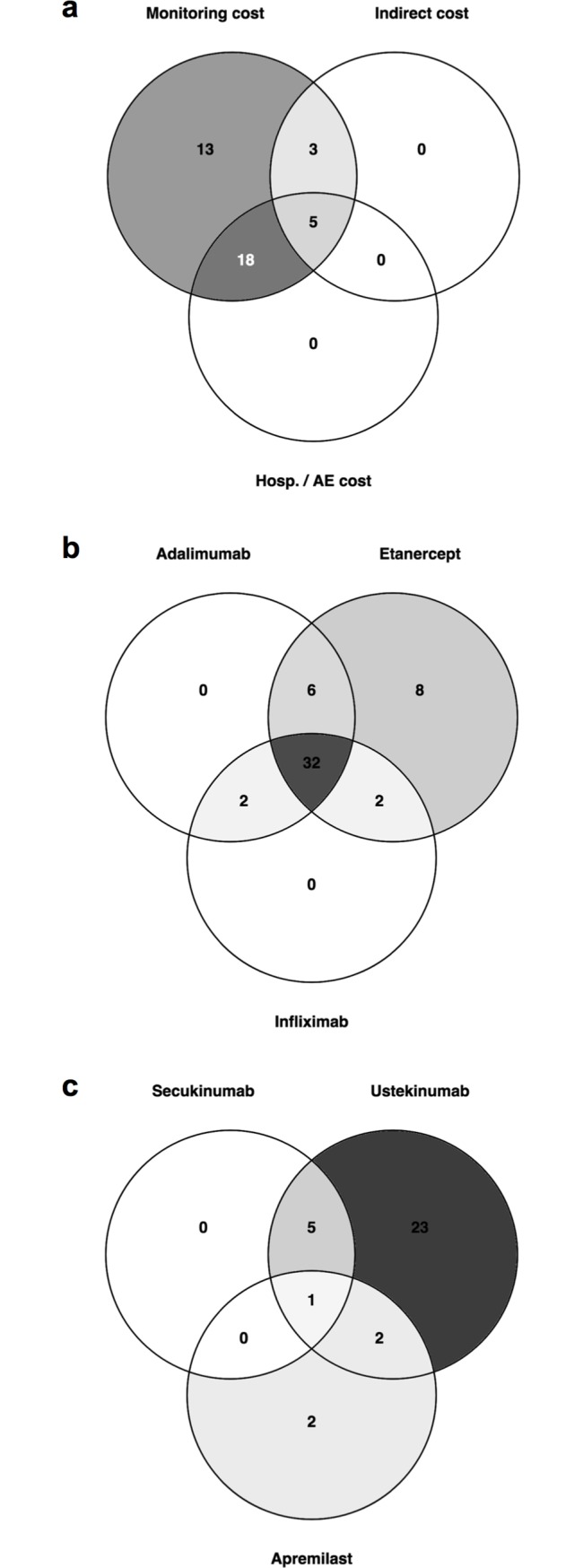
Common characteristics of studies. Depicted are the number of studies sharing included cost elements (a), analyses incorporating TNF-inhibitors (b) and studies integrating ustekinumab, secukinumab and apremilast, which were approved more recently (c). AE: adverse events; Hosp: hospitalization.

Studies were heterogeneous with respect to characteristics and methods (Table 1 and Fig 2; for more details, see S5 Table). Most studies considered a European setting (45%), followed by North American (38%), South American (11%), and Asian (6%) settings. In almost half of the studies, the perspective of the health care system was adopted, followed by a third-party payer (26%) and a societal point of view (13%). A cost-effectiveness analysis was conducted in 55%, a cost-utility analysis in 30%. Five studies reported both cost-effectiveness and cost-utility analyses [43–47]. All but one studies [59] used the PASI response as effectiveness measure. The DLQI was applied in nine analyses [28, 41, 51, 55, 59, 63–65, 75]. QALYs were derived from mapping EQ-5D (European Quality of life in 5 Dimensions, a pre-scored multi-attribute questionnaire to measure health-related quality of life) with PASI or DLQI (n = 9) [30, 51, 54, 55, 59, 65, 73, 74, 77]. National weights were applied in four studies to calculate QALYs [33, 35, 39, 62]. Time trade-off was used in two studies [37, 78].

**Table 1:** Characteristics of included studies.

Characteristics^a^	n (%)	*References*
**Europe**	**24 (45)**	
Spain	7 (13)	*[29, 34, 35, 48, 68, 69, 71]*
Italy	6 (11)	*[33, 37, 39, 41, 74, 76]*
Germany	3 (6)	*[51, 56, 72]*
UK	3 (6)	*[59, 62, 73]*
Sweden	2 (4)	*[38, 55]*
Switzerland	1 (2)	*[49]*
Finland	1 (2)	*[77]*
Czech Rep.	1 (2)	*[54]*
**North America**	**20 (38)**	
USA	17 (32)	*[28, 30–32, 36, 40, 42, 50, 58, 60, 61, 63, 64, 66, 75, 78, 80]*
Canada	3 (6)	*[57, 65, 67]*
**South America**	**6 (11)**	
Brazil	3 (6)	*[44, 45, 70]*
Argentina	1 (2)	*[43]*
Colombia	1 (2)	*[46]*
Venezuela	1 (2)	*[47]*
**Asia**	**3 (6)**	
Japan	2 (4)	*[52, 53]*
Taiwan	1 (2)	*[79]*
**Perspective**		
Health care system	22 (42)	*[33–35, 37, 39, 41, 48–54, 57, 59, 62, 65, 70, 73, 74, 76, 79]*
Third party payer	14 (26)	*[28, 32, 40, 42–47, 61, 64, 66, 72, 75, 80]*
Societal	7 (13)	*[29, 38, 55, 56, 71, 73, 78]*
Not clearly mentioned	11 (21)	*[30, 31, 36, 40, 58, 60, 63, 67–69, 77]*
**Study type**		
CEA	37 (70)	*[28, 29, 31, 32, 34, 36, 38, 40–50, 52, 53, 56, 58, 60, 61, 63, 64, 66–72, 75, 76, 79, 80]*
CUA	21 (40)	*[30, 33, 35, 37, 39, 43–47, 51, 54, 55, 57, 59, 62, 65, 73, 74, 77, 78]*
**Included cost**		
Medication	53 (100)	*[28–80]*
Monitoring^b^	39 (74)	*[28, 30, 33, 35, 37–47, 49, 51, 53–57, 59, 61–66, 70–78, 80]*
Hospitalization	13 (25)	*[30, 35, 37, 39, 51, 54, 55, 59, 62, 70, 71, 73, 74]*
Adverse events^c^	15 (28)	*[39, 43–47, 49, 51, 54, 59, 61, 70, 77, 78, 80]*
Indirect cost^d^	8 (15)	*[30, 38, 53, 55, 56, 71, 73, 78]*
**Comparators**		
Adalimumab	40 (75)	*[28–30, 32–36, 39–41, 43–50, 52, 53, 55–58, 62–64, 67–77, 79]*
Alefacept	11 (21)	*[28, 30, 49, 50, 61, 63, 64, 66, 67, 75, 80]*
Apremilast	5 (9)	*[31–33, 35, 62]*
Efalizumab	10 (19)	*[30, 34, 49, 50, 61, 63, 64, 66, 67, 73]*
Etanercept	48 (91)	*[28–30, 32–37, 39–51, 55–80]*
Infliximab	36 (66)	*[28–30, 32, 34, 36, 39–50, 52, 53, 56–58, 63, 64, 66–70, 72–77]*
Ixekizumab	1 (2)	*[32]*
Secukinumab	6 (11)	*[32, 38, 39, 53, 54, 57]*
Ustekinumab	31 (58)	*[28, 29, 32, 33, 35, 36, 38–40, 44, 46–48, 52–54, 56–58, 60, 65, 67–70, 72, 75–79]*

^a^ When more than one category applied to a study, it was grouped into each appropriate category.

^b^ Monitoring cost included laboratory tests, instrumental procedures such as X-rays, and physician visits.

^c^ Adverse events included, e.g., infections and allergic reactions.

^d^ Indirect cost comprised productivity loss due to absenteeism, presenteeism, and/or unemployment.

n: number of studies; %: percentage of all studies; CEA: cost-effectiveness analysis; CUA: cost-utility analysis; Rep.: Republic; UK: United Kingdom.

On the costing side, all studies included drug costs and 74% considered monitoring costs, i.e. expenditure for laboratory or instrumental tests. The cost evoked by adverse events and hospitalization due to exacerbation and/or for the management of adverse events was incorporated less frequently (25% and 28%, respectively). Indirect cost due to unemployment, sick leave and lower productivity at work was studied in 15%. One third of all studies (n = 18) [35, 37, 39, 43–47, 49, 51, 54, 59, 61, 62, 70, 74, 77, 80] considered monitoring cost and cost for hospitalization and/or adverse events, while the most exhaustive costing side was adopted in five analyses [30, 55, 71, 73, 78] (Fig 2A). Etanercept was included as a comparator in 91% of all analyses, adalimumab in 75%, infliximab in 66%, ustekinumab in 58%, secukinumab in 11%, and apremilast in 9%. Ixekizumab was considered in one study. The majority of studies analyzed the economic relationship between the TNF-inhibitors adalimumab, etanercept, and infliximab (n = 32; 60%) [28–30, 32, 34, 36, 39–41, 43–50, 56–58, 63, 64, 67–70, 72–77] (Fig 2B). In all six studies investigating secukinumab, this medication was compared to ustekinumab (Fig 2C). One of these studies additionally incorporated apremilast [32] (Fig 2C). Most commonly, a Markov model was adapted (40%) [28, 30, 33, 35, 37, 39, 51, 52, 54–59, 62, 65, 70, 73, 77, 78], followed by decision trees (20%) [34, 36, 43–49, 69]. The time horizon of data assessment varied between 10 weeks and 20 years. Most frequently, a time period of 12 weeks (19%) [28, 29, 49, 53, 58, 63, 64, 66, 67, 72], 1 year (34%) [28, 30–32, 38, 40, 42, 50, 52, 53, 58, 60, 67, 69, 71, 75, 76, 79], and 10 years (19%) [37–39, 51, 54, 55, 57, 59, 62, 65] was assumed.

**Fig 3:**
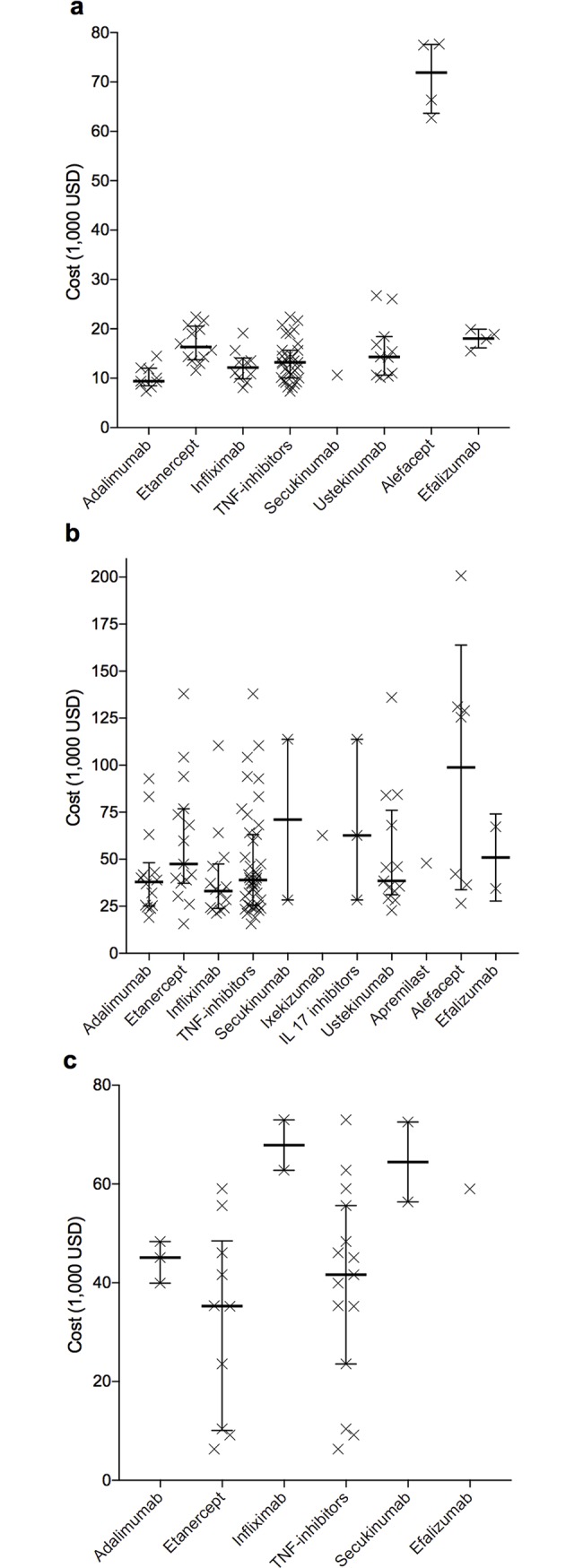
Cost per outcome. Cost per reduction of the Psoriasis Area and Severity Index by 75% (PASI 75 response) was assessed for treatment courses of 12 weeks (a) or one year (b). Part c shows cost per Quality-Adjusted Life Year (QALY). Each x represents one study result. IL 17: interleukin 17; TNF: tumor necrosis factor. Bars: medians; vertical lines: interquartile ranges.

Table 2 shows the results of the studies included as cost per PASI 75 response, cost per DLQI MID, and cost per QALY, compared to non-biological therapy or placebo (for details, see S6 Table). Diversity in the study design and methodology resulted in broad intervals with distinct overlap when comparing biologicals with each other (see Fig 3A–3C for cost per PASI 75 response for 12 weeks and 1 year, and cost per QALY). Calculation of Pearson’s correlation coefficients for cost/PASI, cost/DLQI, and cost/QALY revealed no significant correlation.

**Table 2:** Summary of economic findings.

Biological	Cost per PASI 75^a^	*References*	Cost per DLQI MID^a^	*References*	Cost per QALY^a^	*Referen-ces*
**Adalimumab**	7,325–92,871	*[28, 29, 32, 34, 36, 40, 48–50, 52, 53, 56, 58, 63, 64, 67–70, 72, 75, 76, 79]*	3,655–26,871	*[28, 64, 75]*	39,952–48,341	*[55, 73, 74]*
**Alefacept**	36,430–200,734	*[28, 42, 49, 50, 61, 63, 64, 66, 67, 75, 80]*	28,167–155,255	*[28, 63, 64, 75]*	NR	*NA*
**Apremilast**^**b**^	47,960–157,309	*[31, 32]*	NR	*NA*	NR	*NA*
**Efalizumab**	15,524–78,937	*[34, 49, 50, 61, 63, 64, 66, 67]*	5,478–6,831	*[63, 64]*	59,009	*[73]*
**Etanercept**	11,590–138,009	*[28, 29, 32, 34, 36, 37, 40, 42, 48–51, 56, 58, 60, 61, 63, 64, 66–70, 72, 75, 76, 79, 80]*	2,342–44,796	*[28, 63, 64, 75]*	6,347–59,069	*[37, 51, 55, 59, 73, 74]*
**Infliximab**	8,077–229,392	*[28, 29, 32, 34, 36, 40, 42, 48–50, 52, 53, 56, 58, 63, 64, 66–70, 72, 75, 76]*	3,652–11,348	*[28, 63, 64, 75]*	62,767–73,021	*[73, 74]*
**Ixekizumab**	62,707	*[32]*				
**Ustekinumab**	10,151–136,075	*[28, 29, 32, 36, 38, 40, 48, 52, 53, 56, 58, 60, 67–70, 72, 75, 76, 79]*	15,500–32,144	*[28, 75]*	NR	*NA*
**Secukinumab**	10,654–113,858	*[32, 38, 53]*	NR	*NA*	56,380–72,544	*[39, 57]*

^a^ Cost per outcome in USD is presented as compared to non-biologic therapy or placebo. Incremental analyses results comparing two biologicals are not included in this table. In addition, studies evaluating treatment sequences are not considered.

^b^ Apremilast was compared to methotrexate.

DLQI MID: minimal important difference in the Dermatology Life Quality Index; NA: not assessed; NR: not reported; PASI 75: reduction of the Psoriasis Area and Severity Index by 75%; QALY: Quality-Adjusted Life Year.

Pairwise comparison of biologicals led to partially conflicting results, since some studies concluded that one biological was dominant over another, while other studies concluded the opposite (Table 3). The category containing the largest number of studies within one pairwise comparison was assumed to reflect the economic relationship most accurately. Adalimumab was found to cost less per treatment success in comparison with etanercept (53.3% of all studies), infliximab (58.3%), ustekinumab (57.9%), secukinumab (66.7%), alefacept (100%), and efalizumab (85.7%). Etanercept was more cost-effective than alefacept (81.8%) and efalizumab (62.5%) but less cost-effective compared with infliximab (57.7%) and ustekinumab (57.1%). The economic relationship between etanercept and secukinumab remains unclear, as one study identified etanercept as more cost-effective, whereas according to another study secukinumab provided higher benefit at higher cost. Infliximab possessed higher cost-effectiveness compared with alefacept (88.9%) and efalizumab (71.4%) and lower cost-effectiveness compared with ustekinumab (42.1%). Results of comparisons between infliximab and secukinumab were conflicting (higher cost-effectiveness and lower cost-effectiveness in one study each). Ustekinumab was superior to alefacept (100%) and efalizumab (100%); however, it was less cost-effective than secukinumab (40%). Alefacept was less cost-effective compared with efalizumab (83.3%).

**Table 3:** Pairwise comparisons of cost-effectiveness of biologicals.

Ac-tive treat-ment ↓	Comparator to treatment →	ETA	INX	UST	SEC	ALE	EFA
	Economic relationship between treatment and comparator ↓	n (%); *references*	n (%); *references*	n (%); *references*	n (%); *references*	n (%); *references*	n (%); *references*
**ADA**	Treatment dominant	2 (6.7); *[70, 73]*	1 (4.2); *[70]*	NR	NR	NR	1 (14.3); *[73]*
	Treatment dominated	5 (16.7); *[43–47]*	NR	1 (5.3); *[56]*	NR	NR	NR
	Higher benefit at higher cost	2 (6.7); *[30, 55]*	NR	NR	NR	NR	NR
	Lower benefit at lower cost^a^	NR	2 (8.3); *[41, 73]*	2 (10.5); *[70, 76]*	1 (33.3); *[39]*	NR	NR
	More cost-effective	**16 (53.3);** *[28, 29, 34, 36, 40, 48, 56, 58, 63, 64, 67–69, 72, 75, 79]*	**14 (58.3);** *[34, 36, 40, 48–50, 52, 53, 56, 58, 63, 67, 68, 74]*	**11 (57.9);** *[28, 29, 36, 40, 48, 53, 56, 58, 67, 72, 79]*	**2 (66.7);** *[32, 53]*	**7 (100);** *[28, 49, 50, 63, 64, 67, 75]*	**6 (85.7);** *[34, 49, 50, 63, 64, 67]*
	Equally cost-effective	1 (3.3); *[71]*	NR	1 (5.3); *[68]*	NR	NR	NR
	Less cost-effective	4 (13.3); *[32, 50, 55, 74]*	7 (29.2); *[28, 29, 32, 64, 69, 72, 75]*	4 (21.1); *[32, 52, 69, 75]*	NR	NR	NR
**ETA**	Treatment dominant	NA	5 (19.2); *[43–47]*	3 (14.3); *[44, 46, 47]*	NR	2 (18.2); *[63, 80]*	1 (12.5); *[63]*
	Treatment dominated	NA	NR	4 (19); *[56, 65, 76, 78]*	NR	NR	NR
	Higher benefit at higher cost	NA	NR	NR	NR	NR	NR
	Lower benefit at lower cost^a^	NA	2 (7.7); *[41, 64]*	NR	1 (50)^b^; *[39]*	NR	NR
	More cost-effective	NA	4 (15.4); *[34, 48, 50, 74]*	2 (9.5); *[32, 48]*	1 (50)^b^; [32]	**9 (81.8);** *[28, 42, 49, 50, 61, 64, 66, 67, 75]*	**5 (62.5);** *[34, 50, 61, 64, 66]*
	Equally cost-effective	NA	NR	NR	NR	NR	1 (12.5); *[67]*
	Less cost-effective	NA	**15 (57.7);** *[28, 29, 32, 36, 40, 42, 49, 58, 63, 66–69, 72, 75]*	**12 (57.1);** *[28, 29, 36, 40, 58, 60, 67–69, 72, 75, 79]*	NR	NR	1 (12.5); *[49]*
**INX**	Treatment dominant	NR	NA	NR	NR	NR	NR
	Treatment dominated	See above	NA	2 (10.5); *[39, 76]*	NR	NR	NR
	Higher benefit at higher cost	See above	NA	2 (10.5); *[56, 57]*	NR	1 (11.1); *[64]*	2 (28.6); *[30, 64]*
	Lower benefit at lower cost^a^	NR	NA	NR	NR	NR	NR
	More cost-effective	See above	NA	6 (31.6); *[28, 29, 32, 58, 72, 75]*	1 (50)^c^; *[32]*	**8 (88.9);** *[28, 42, 49, 50, 63, 66, 67, 75]*	**5 (71.4);** *[49, 50, 63, 66, 67]*
	Equally cost-effective	NR	NA	1 (5.3); *[40]*	NR	NR	NR
	Less cost-effective	See above	NA	**8 (42.1);** *[36, 48, 49, 52, 53, 67–69]*	1 (50)^c^; [53]	NR	NR
**UST**	Treatment dominant	See above	See above	NA	NR	NR	NR
	Treatment dominated	See above	NR	NA	1 (20); *[54]*	NR	NR
	Higher benefit at higher cost	NR	NR	NA		NR	NR
	Lower benefit at lower cost^a^	NR	See above	NA	1 (20); *[39]*	NR	NR
	More cost-effective	See above	See above	NA	1 (20); *[32]*	**3 (100);** *[28, 67, 75]*	**1 (100);** *[67]*
	Equally cost-effective	NR	See above	NA	NR	NR	NR
	Less cost-effective	See above	See above	NA	**2 (40); *[38, 53]***	NR	NR
**ALE**	Treatment dominant	NR	NR	NR	NR	NA	NR
	Treatment dominated	See above	NR	NR	NR	NA	NR
	Higher benefit at higher cost	NR	NR	NR	NR	NA	NR
	Lower benefit at lower cost^a^	NR	See above	NR	NR	NA	NR
	More cost-effective	NR	NR	NR	NR	NA	1 (16.7); *[61]*
	Equally cost-effective	NR	NR	NR	NR	NA	NR
	Less cost-effective	See above	See above	See above	NR	NA	**5 (83.3);** *[49, 50, 63, 64, 66]*

The terms “treatment dominant”, “treatment dominated”, “higher benefit at higher cost”, and “lower benefit at lower cost” refer to direct comparisons between biologicals in terms of incremental cost-effectiveness ratios. “Treatment dominant” means that the active treatment was more effective and less costly than the comparator while “treatment dominated” means that the active treatment was less effective but more costly than the comparator. When biologicals were not compared directly with each other but with non-biological therapy or placebo, the biological that costed less per benefit was considered “more cost-effective”. “Equally” and “less cost-effective” refer to the same or a higher cost per benefit, respectively (for further explanations, see Methods). When both PASI and DLQI MID were provided, PASI was considered for better inter-study comparison. When more than one dosing regime was given, the most cost-effective was considered. Comparisons of treatment sequences were not included. Apremilast and ixekizumab were analyzed in merely one study; therefore, they were not included into the table. If more than one ICER was reported (e.g., ICER 1: drug A vs. B, ICER 2: drug A vs. C), the study appears both in the appropriate category for pairwise comparison between drug A and drug B and in the appropriate category for pairwise comparison between drug A and drug C.

^a^ “Lower benefit at lower cost” means that the comparator of the active treatment yields higher benefit at higher cost.

^b^ Data on the economic relationship between etanercept and secukinumab are conflicting with one study stating that etanercept was more cost-effective and another study showing lower benefit at lower cost compared to secukinumab.

^c^ Infliximab (INF) was found to be more cost-effective compared to secukinumab (SEC) in one study and less cost-effective in another analysis. Therefore, the economic relationship remains unclear.

ADA: adalimumab; ETA: etanercept; INX: infliximab; UST: ustekinumab; SEC: secukinumab; ALE: alefacept; EFA: efalizumab; n: number of studies; NA: not applicable; NR: not reported; %: percentage of all studies investigating the economic relationship between treatment and comparator. Bold numbers indicate the highest percentage as proxy for the most accurate economic relationship.

In order to summarize the results of Table 3, the economic category with the highest share was extracted to Table 4 as it was assumed to reflect the economic relationship between two biologicals most accurately. For example, a majority of 53.3% of studies investigating the relationship between adalimumab and etanercept found that adalimumab was more cost-effective. This is illustrated by the up arrow in Table 4.

**Table 4:** Summary of pairwise comparisons.

Comparator →	ADA	ETA	INX	UST	SEC	ALE	EFA	% (more cost-effective / all comparators)
Active treatment								
↓								
**ADA**	NA	↑	↑	↑	↑	↑	↑	100^a^
**ETA**	**↓**	NA	**↓**	**↓**	→	↑	↑	40
**INX**	**↓**	↑	NA	**↓**	→	↑	↑	60
**UST**	**↓**	↑	↑	NA	**↓**	↑	↑	66.7
**SEC**	**↓**	→	→	↑	NA	NR	NR	50
**ALE**	**↓**	**↓**	**↓**	**↓**	NR	NA	**↓**	0
**EFA**	**↓**	**↓**	**↓**	**↓**	NR	↑	NA	20

↑Active treatment was more cost-effective in the largest proportion of studies investigating this pairwise comparison (see Table 3, numbers highlighted in bold). ↓ Active treatment was less cost-effective according to most studies. →The economic relationship between the active treatment and the comparator remains unclear due to conflicting study results.

^a^ This does not mean that adalimumab (ADA) was economically superior in 100% of *all studies* containing pairwise comparisons, because only the economic category with the majority of studies was extracted from Table 3 into Table 4 as an approximation for the most reliable economic relationship.

ADA: adalimumab; ETA: etanercept; INX: infliximab; UST: ustekinumab; SEC: secukinumab; ALE: alefacept; EFA: efalizumab; %: percentage; NA: not applicable; NR: not reported.

Overall, adalimumab was superior to its comparators most frequently, i.e., in aggregated data from all pairwise comparisons (100%), followed by ustekinumab (66.7%; 4 of 6 comparisons), infliximab (60%; 3 of 5 comparisons), secukinumab (50%; 1 of 2 comparisons), etanercept (40%; 2 of 5 comparisons), efalizumab (20%, 1 of 5 comparisons), and alefacept (0%; 0 of 5 comparisons; Table 4).

When summarizing study conclusions, 75% of all studies investigating secukinumab favored this drug. Apremilast was preferred in 60%, adalimumab in 40%, etanercept in 37.5%, ustekinumab in 32.3%, and infliximab in 30.6%. Ixekizumab, alefacept and efalizumab were not favored in the conclusions of any study. However, ixekizumab was only considered in one study, whereas alefacept was included in 11 and efalizumab in 10 studies (Table 5). Further information on comparators integrated into the studies is presented in S7 Table.

**Table 5:** Summary of study conclusions.

Study conclusions were in favor of. . .		
Biological	n / n (%)	*References*
Adalimumab	16 / 40 (40)	*[30, 34, 36, 40, 48–50, 53, 58, 63, 64, 67, 68, 70, 73, 79]*
Alefacept	0 / 11 (0)	*NA*
Apremilast	3 / 5 (60)	*[33, 35, 62]*
Efalizumab	0 /10 (0)	*NA*
Etanercept	18 / 48 (37.5)	*[37, 43–47, 50, 51, 55, 59, 74, 80]*
Infliximab	11 / 36 (30.6)	*[28, 29, 32, 41, 42, 49, 56, 64, 66, 72, 75]*
Ixekizumab	0 / 1 (0)	*NA*
Secukinumab	4 / 6 (66.7)	*[38, 39, 54, 57]*
Ustekinumab	10 / 31 (32.3)	*[52, 56, 60, 65, 68, 69, 76–79]*

n / n: number of studies which were in favor of the biological / number of all studies addressing the respective biological; %: percentage; NA: not applicable.

Results of pairwise comparisons (Table 4) and study conclusions (Table 5) were sometimes discrepant due to different methods of aggregating data. For example, adalimumab was preferred in aggregated data from all pairwise comparisons (Table 4) but study conclusions favored adalimumab only in 40% of the studies (Table 5). Studies evaluating treatment sequences as decision trees or by Markov modeling did not allow one-by-one comparison of biologicals. Nevertheless, the authors drew conclusions regarding cost-effectiveness. Therefore, these studies were included into Table 5 but not into Table 4. The verbal conclusion of the authors depicted in Table 5 could be influenced by assumed willingness-to-pay thresholds, whereas the summary of pairwise comparisons (Table 4) aggregates the economic relationship between individual biologicals based on actual data stated in the results section of included studies.

**Fig 4:**
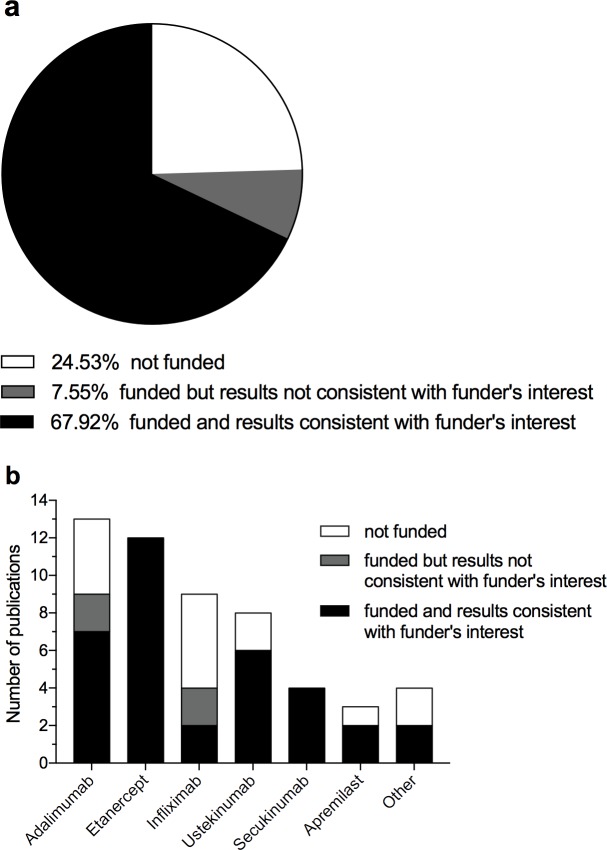
Funding information. In 36 studies funding went congruently with observed outcome. Four studies were funded but the results were not consistent with the funder’s financial interest. Thirteen studies were not funded (a). When stratifying according to individual biologicals high consistency between funding and observed outcome could be detected for all biologicals except for infliximab (b). Other: Two studies favored at least two biologicals, one funded study detected lacking cost-effectiveness of a competitor’s biological (i.e. congruent to funder’s interest), and one study found lacking cost-effectiveness of all biologicals when compared to the funder’s topical therapy (i.e. congruent to funder’s interest).

Three-quarters of all studies [28, 31–35, 37–39, 43–60, 62–67, 69, 73–75, 77, 78, 80] were financially supported, either by direct funding or by contribution of employees of pharmaceutical companies as authors (for details on funding information, see S8 Table). In 90% of these [28, 31–35, 37–39, 43–60, 62, 65, 67, 69, 73, 74, 77, 78, 80], funding for a study went congruently with observed outcome, meaning that the funder’s biological was considered most cost-effective or provided additional benefit at acceptable costs (Fig 4A). Stratification of funding according to individual biologicals is shown in Fig 4B.

## Discussion

To our knowledge, this is the first systematic review on economic evaluations of biologicals for treatment of psoriasis which includes pairwise comparisons and recently approved biologicals or small molecules. Synthesis of cost-effectiveness resulted in enormous intervals with distinct overlap, precluding meaningful comparison between biologicals. When PASI response was adopted as outcome measure, cost ranges were larger than with DLQI or QALY as outcome measures. It can be speculated that variations observed for DLQI or QALY were smaller because fewer studies incorporated these outcome parameters. In pairwise comparisons, adalimumab seemed to be most cost-effective, followed by ustekinumab, infliximab, secukinumab and etanercept, while alefacept and efalizumab were least cost-effective. When evaluating study conclusions, the newly approved drugs secukinumab and apremilast were favored, followed by adalimumab, etanercept, ustekinumab, and infliximab. Alefacept, efalizumab, and ixekizumab were not preferred in any of the study conclusions.

Several findings of our systematic review are consistent with previously published reviews. Hamilton and colleagues [17] examined economic data for all treatment options approved for psoriasis. They found a wide range of costs and outcomes as well as high levels of uncertainty. However, pairwise comparisons were not performed. Zhang and colleagues [16] reviewed all treatment options for psoriasis, focusing on the evaluation of quality of studies and drivers of cost-effectiveness. They detected overall low quality standards and failed to identify a single most cost-effective agent. Two Canadian health technology assessments [18, 19] evaluated biologicals clinically and economically from a national perspective. However, no synthesis or recommendation was given and conclusions were indistinct. A recent systematic literature search on systemic treatments for psoriasis focused on study characteristics and detected heterogeneous study designs as well [15].

Integration of cost-effectiveness data into the context of other systemic and non-systemic treatment options is crucial for clinicians and policy-makers in order to identify an optimal treatment sequence. Some of the studies included into this review compared small molecules and/or biologicals with non-biological therapies (e.g., [31]). There are several reviews on the economic evaluation of all systemic treatments or even all treatment options for psoriasis [15–17], but further analyses comprising a comprehensive collection of treatments for moderate-to-severe psoriasis in clinical routine are required.

A strength of this review is the methodology of analyzing the economic relationship between biologicals in pairwise comparisons. By this way, the analysis does not rely on pure numbers which are prone to heterogeneity in assumptions and designs but allows for identifying a rank order of cost-effectiveness within individual studies. The categorization of studies by solely extracting whether biological A is “economically better”, “similar”, or “worse” compared to biological B enables to include studies whose absolute cost-effectiveness results vary significantly due to different model assumptions. On the other side, this review cannot quantify the difference in cost-effectiveness between biologicals in precise numbers, because no absolute cost-per-outcome data was extracted. A further strength is inclusion of publications investigating long-term cost-effectiveness which is essential for treatment of lifelong chronic diseases such as psoriasis.

Our review comprised abstracts without full-text articles in order to capture data on recently approved biologicals. This allowed us to include economic data on apremilast and secukinumab for the first time. Moreover, consideration of abstracts and posters broadened the perspective and facilitated integration of economic data from a large number of countries. However, information provided in abstracts and on posters was limited, resulting in a lower quality of data.

Several limitations have to be considered when interpreting our results. First, the findings may be influenced by publication bias, as analyses with insignificant results tend to remain unpublished. Merely one study included into our review detected no significant differences in cost and effect when comparing two biologicals. Therefore, differences in cost-effectiveness between biologicals may be sometimes overestimated in this review.

Second, cost-effectiveness findings are affected by model assumptions, sometimes even resulting in contrary conclusions, as presented in Table 3. The following factors may contribute to this high variance:

- Cost elements: Differences between biologicals in frequency of physician visits, laboratory tests, adverse events, and hospitalization (due to non-response or management of adverse events) and the associated cost, especially for hospitalization, result in high variance between different studies [16].
- Outcome assumptions: The choice of outcome parameters (PASI, DLQI, or QALY) leads to different cost effectiveness findings (see Table 2 and Fig 3). For example, ixekizumab and secukinumab have PASI 75 response rates comparable to that of ustekinumab, but significantly higher PASI 90 response rates. Consequently, cost-effectiveness results change with the choice of outcome parameters. Moreover, the choice of efficacy data and the method of synthesis differed between included studies.
- Perspective of analysis: Ustekinumab is administered every 12 weeks subcutaneously by the patient at home while infliximab is given every 8 weeks intravenously in a hospital or practice. If a societal perspective is adopted expenditure for travelling and lost productivity of patients add up to the costing side. Moreover, unit prices for medication and administration differ between countries.
- Time horizon: Several biologicals are initially administered in higher dosages and/or at shorter intervals. In the successive treatment course fewer medication units are required. Thus, initial additional costs dilute when applying a long time horizon.
- Type of model: Models of included studies varied from simply dividing cost arising in a defined period of time by the effectiveness at the end of this time to Markov models or decision trees. The definition of a treatment sequence thereby alters cost and effectiveness outcomes.

Zhang and colleagues studied these factors extensively in the context of psoriasis treatment and identified treatment cost, hospitalization, efficacy assumptions, utility valuation, time horizon, and model structure as key drivers of cost effectiveness [16]. Third, QALYs assessed by cost-utility analyses were mostly derived from mapping PASI response and/or DLQI scores with EQ-5D responses. However, previous publications reported only a weak to moderate correlation between these clinical outcomes and the EQ-5D [81–83], which could lead to systematic error.

Finally, since the majority of included studies were sponsored by the pharmaceutical industry and their results were in line with the funder's interest, estimations of cost-effectiveness have to be interpreted with caution.

In conclusion, this systematic review provides an actual overview on economic evaluations of biologicals including pairwise comparisons, but also highlights limitations and gaps in health economic evidence and the need to address the following issues:

- Future analyses should establish a comprehensive *costing side*, including expenditure for medication, screening, monitoring, delivery, hospitalization, and management of adverse events and comorbidities. If a societal perspective is adopted, cost due to productivity loss in terms of presenteeism, absenteeism, and unemployment should be included.
- On the *outcome side* reliable measures (PASI, DLQI, or QALY) should be adopted. Despite the QALY’s favorable property of enabling comparison of cost-effectiveness across diseases, its use in dermatology is problematic, as life expectancy is not dramatically lowered due to chronic inflammatory dermatoses. Moreover, derivation of QALYs can be biased as described above. PASI 75 response, a validated instrument commonly used in clinical studies on psoriasis, provides a more objective view. If the PASI response is adopted as outcome measure for all biologicals, a median cost per response can be calculated and compared across this group. This approach can be helpful to guide price determination for biosimilars and newly approved biologicals.
- Analyses should consider *all currently approved biologicals* and reasonable treatment alternatives from a clinical point of view, provide a sufficiently long time horizon (i.e., at least several years) to reflect unpredictable disease progression and secondary treatment failure, and account for non-adherence.
- Study assumptions and results should be *reported clearly* with respect to population, intervention, comparator, cost and outcome assumptions, perspective, and generalizability.

Incorporating these aspects can help to increase our comprehension of cost-effectiveness of biologicals for psoriasis in a real-life setting and thereby assist physicians and policy-makers in responsibly allocating health care resources.

## Supporting information

S1 Fig Search strategy. All keywords in the concepts are connected with “or”. The search strategy was limited to articles in the English, German, and Spanish language. For detailed string terms see S2 Table.
(TIFF)

S1 Table PRISMA checklist. (DOC)

S2 Table Search strategy. (DOCX)

S3 Table Quality assessment checklist. (DOCX)

S4 Table Quality assessment. (DOCX)

S5 Table Study characteristics and methods. (DOCX)

S6 Table Economic results of included studies. (DOCX)

S7 Table Comparators to favored biologicals according to study conclusions. (DOCX)

S8 Table Funding information. (DOCX)
