# Supplementary figures and images for: Biologicals and small molecules in psoriasis: A systematic review of economic evaluations
Source: PLoS One. 2018 Jan 3;13(1):e0189765. doi: 10.1371/journal.pone.0189765 (PMC5751984; doi:10.1371/journal.pone.0189765)

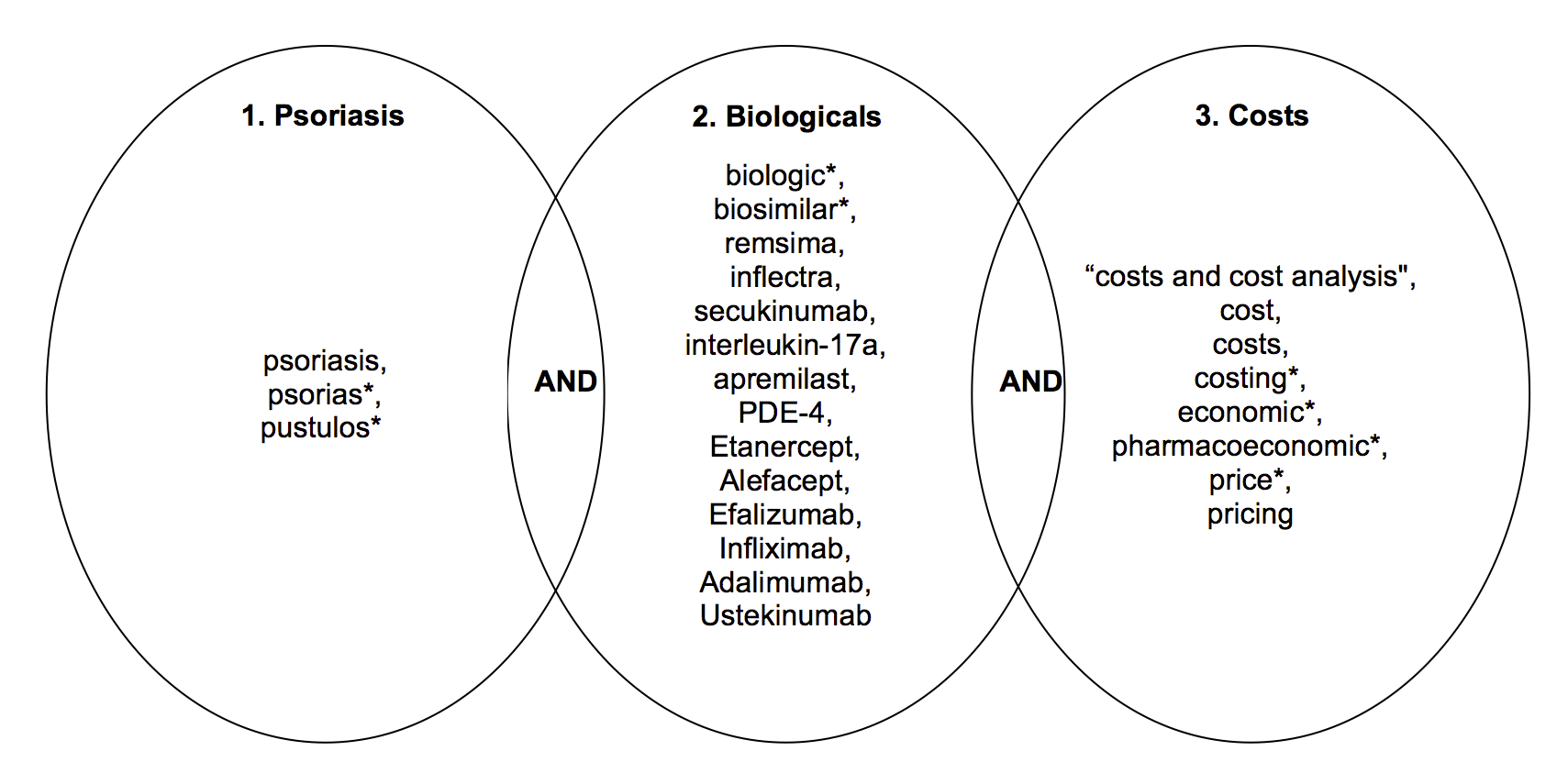

Supplement: S1 Fig — All keywords in the concepts are connected with “or”. The search strategy was limited to articles in the English, German, and Spanish language. For detailed string terms see S2 Table. (TIFF) [file pone.0189765.s001.tiff]
