# Supplementary material for: Biologicals and small molecules in psoriasis: A systematic review of economic evaluations
Source: PLoS One. 2018 Jan 3;13(1):e0189765. doi: 10.1371/journal.pone.0189765 (PMC5751984; doi:10.1371/journal.pone.0189765)
Supplement: S2 Table — (DOCX) [file pone.0189765.s003.docx]

## S2 Table. Search strategy.

| **PubMed:**  (psoriasis[mh:noexp] OR psorias*[tw] OR pustulos*[tw]) AND (biologic*[tw] OR biosimilar*[tw] OR remsima[tw] OR inflectra[tw] OR secukinumab[tw] OR interleukin-17a[tw] OR apremilast[tw] OR PDE-4[tw] OR Etanercept[tw] OR Alefacept[tw] OR Efalizumab[tw] OR Infliximab[tw] OR Adalimumab[tw] OR Ustekinumab[tw]) AND ("costs and cost analysis"[mh] OR cost[tw] OR costs[tw] OR costing*[tw] OR economic*[tw] OR pharmacoeconomic*[tw] OR price*[tw] OR pricing[tw]) AND (english[lang] OR spanish[lang] OR german[lang]) |
| --- |
| **Cochrane:**  Search: Title, Abstract, Keywords (psorias* OR pustulos*) AND (biologic* OR biosimilar* OR remsima OR inflectra OR secukinumab OR interleukin-17a OR apremilast OR PDE-4 OR Etanercept OR Alefacept OR Efalizumab OR Infliximab OR Adalimumab OR Ustekinumab) AND (cost OR costs OR costing* OR economic* OR pharmacoeconomic* OR price* OR pricing) |
| **EconLit:**  Advanced Search: TI OR AB OR SU (psorias* OR pustulos*) AND (biologic* OR biosimilar* OR remsima OR inflectra OR secukinumab OR interleukin-17a OR apremilast OR PDE-4 OR Etanercept OR Alefacept OR Efalizumab OR Infliximab OR Adalimumab OR Ustekinumab) AND (cost OR costs OR costing* OR economic* OR pharmacoeconomic* OR price* OR pricing) |
| **Lilacs:**  Search: Title, abstract, subject (psorias$ OR pustulos$) AND (biologic$ OR biosimilar$ OR remsima OR inflectra OR secukinumab OR interleukin-17a OR apremilast OR PDE-4 OR Etanercept OR Alefacept OR Efalizumab OR Infliximab OR Adalimumab OR Ustekinumab) AND (cost OR costs OR costing$ OR economic$ OR pharmacoeconomic$ OR price$ OR pricing) |
