# Supplementary material for: Biologicals and small molecules in psoriasis: A systematic review of economic evaluations
Source: PLoS One. 2018 Jan 3;13(1):e0189765. doi: 10.1371/journal.pone.0189765 (PMC5751984; doi:10.1371/journal.pone.0189765)
Supplement: S3 Table — (DOCX) [file pone.0189765.s004.docx]

## S3 Table. Quality assessment checklist.

| **Checklist item^a^** | **n (%)^b^** |
| --- | --- |
| **Study design** |  |
| 1. Was the research question stated? | 53 (100) |
| 1. Was the economic importance of the research question stated? | 38 (72) |
| 1. Was/were the viewpoint(s) of the analysis clearly stated and justified? | 41 (77) |
| 1. Was a rationale reported for the choice of the alternative programmes or interventions compared? | 45 (85) |
| 1. Were the alternatives being compared clearly described? | 49 (92) |
| 1. Was the form of economic evaluation stated? | 53 (100) |
| 1. Was the choice of form of economic evaluation justified in relation to the questions addressed? | 8 (15) |
| **Data collection** |  |
| 1. Was/were the source(s) of effectiveness estimates used stated? | 47 (89) |
| 1. Were details of the design and results of the effectiveness study given (if based on a single study)? | 8 (15) |
| 1. Were details of the methods of synthesis or meta-analysis of estimates given (if based on an overview of several effectiveness studies)? | 21 (40) |
| 1. Were the primary outcome measure(s) for the economic evaluation clearly stated? | 51 (96) |
| 1. Were the methods used to value health states and other benefits stated? | 19 (36) |
| 1. Were the details of the subjects from whom valuations were obtained given? | 4 (8) |
| 1. Were productivity changes (if included) reported separately? | 8 (15) |
| 1. Was the relevance of productivity changes to the study question discussed? | 18 (34) |
| 1. Were quantities of resources reported separately from their unit cost? | 31 (58) |
| 1. Were the methods for the estimation of quantities and unit costs described? | 51 (96) |
| 1. Were currency and price data recorded? | 53 (100) |
| 1. Were details of price adjustments for inflation or currency conversion given? | 12 (23) |
| 1. Were details of any model used given? | 30 (57) |
| 1. Was there a justification for the choice of model used and the key parameters on which it was based? | 6 (11) |
| **Analysis and interpretation of results** |  |
| 1. Was time horizon of cost and benefits stated? | 52 (98) |
| 1. Was the discount rate stated? | 21 (40) |
| 1. Was the choice of rate justified? | 6 (11) |
| 1. Was an explanation given if cost or benefits were not discounted? | 3 (6) |
| 1. Were details of statistical test(s) and confidence intervals given for stochastic data? | 30 (57) |
| 1. Was the approach to sensitivity analysis described? | 42 (79) |
| 1. Was the choice of variables for sensitivity analysis justified? | 15 (28) |
| 1. Were the ranges over which the parameters were varied stated? | 33 (62) |
| 1. Were relevant alternatives compared? (i.e. Were appropriate comparisons made when conducting the incremental analysis?) | 48 (91) |
| 1. Was an incremental analysis reported? | 36 (68) |
| 1. Were major outcomes presented in a disaggregated as well as aggregated form? | 36 (68) |
| 1. Was the answer to the study question given? | 53 (100) |
| 1. Did conclusions follow from the data reported? | 53 (100) |
| 1. Were conclusions accompanied by the appropriate caveats? | 40 (75) |
| 1. Were generalizability issues addressed? | 8 (15) |

^a^ Checklist items proposed by the Centre for Reviews and Dissemination guidance for undertaking systematic reviews [22], ^b^ Number (n) and percentage (%) of studies which sufficiently met the corresponding item.
