# Supplementary material for: Biologicals and small molecules in psoriasis: A systematic review of economic evaluations
Source: PLoS One. 2018 Jan 3;13(1):e0189765. doi: 10.1371/journal.pone.0189765 (PMC5751984; doi:10.1371/journal.pone.0189765)
Supplement: S4 Table — (DOCX) [file pone.0189765.s005.docx]

## S4 Table. Quality assessment.

| **Checklist item^a^**  **Author, yr** | **Study design** | | | | | | | **Data collection** | | | | | | | | | | | | | | **Analysis and interpretation of results** | | | | | | | | | | | | | | |
| --- | --- | --- | --- | --- | --- | --- | --- | --- | --- | --- | --- | --- | --- | --- | --- | --- | --- | --- | --- | --- | --- | --- | --- | --- | --- | --- | --- | --- | --- | --- | --- | --- | --- | --- | --- | --- |
|  | **1** | **2** | **3** | **4** | **5** | **6** | **7** | **8** | **9** | **10** | **11** | **12** | **13** | **14** | **15** | **16** | **17** | **18** | **19** | **20** | **21** | **22** | **23** | **24** | **25** | **26** | **27** | **28** | **29** | **30** | **31** | **32** | **33** | **34** | **35** | **36** |
| Ahn, 2013 [28] | ✔ | ✔ | ✔ | NA | ✔ | ✔ | ✔ | ✔ | NA | ✔ | ✔ | NA | NA | NA | no | ✔ | ✔ | ✔ | no | NA | NA | ✔ | NA | NA | no | ✔ | ✔ | ✔ | ✔ | ✔ | ✔ | ✔ | ✔ | ✔ | ✔ | NA |
| Alfageme Roldán, 2016 [29] | ✔ | ✔ | ✔ | ✔ | ✔ | ✔ | no | ✔ | NA | no | ✔ | ✔ | no | ✔ | ✔ | ✔ | ✔ | ✔ | no | no | no | ✔ | NA | NA | no | no | no | no | no | ✔ | ✔ | no | ✔ | ✔ | ✔ | no |
| Anis, 2011 [30] | ✔ | ✔ | no | NA | ✔ | ✔ | ✔ | ✔ | NA | ✔ | ✔ | ✔ | ✔ | ✔ | ✔ | no | ✔ | ✔ | no | NA | NA | ✔ | NA | NA | no | ✔ | ✔ | no | ✔ | ✔ | ✔ | ✔ | ✔ | ✔ | ✔ | NA |
| Armstrong, 2015 [31] | ✔ | no | no | ✔ | no | ✔ | no | ✔ | NA | ✔ | no | No | NA | NA | no | no | no | ✔ | no | no | no | ✔ | NA | NA | no | ✔ | no | NA | NA | ✔ | ✔ | no | ✔ | ✔ | no | no |
| Asche, 2017 [32] | ✔ | ✔ | ✔ | ✔ | ✔ | ✔ | ✔ | ✔ | NA | no | ✔ | ✔ | no | NA | no | ✔ | no | ✔ | no | ✔ | ✔ | ✔ | no | no | no | no | ✔ | no | ✔ | ✔ | ✔ | ✔ | ✔ | ✔ | ✔ | no |
| Barbieri, 2015 [33] | ✔ | no | ✔ | ✔ | ✔ | ✔ | no | ✔ | NA | ✔ | ✔ | ✔ | no | NA | no | no | ✔ | ✔ | no | ✔ | no | ✔ | ✔ | no | NA | ✔ | ✔ | no | ✔ | ✔ | ✔ | ✔ | ✔ | ✔ | no | NA |
| Blasco, 2009 [34] | ✔ | ✔ | ✔ | ✔ | ✔ | ✔ | no | ✔ | NA | ✔ | ✔ | No | NA | NA | no | ✔ | ✔ | ✔ | no | ✔ | no | ✔ | NA | NA | no | ✔ | ✔ | no | ✔ | ✔ | ✔ | ✔ | ✔ | ✔ | ✔ | NA |
| Carrascosa, 2015 [35] | ✔ | no | ✔ | ✔ | ✔ | ✔ | no | ✔ | NA | ✔ | ✔ | ✔ | no | NA | no | ✔ | ✔ | ✔ | no | ✔ | no | ✔ | ✔ | ✔ | NA | no | ✔ | no | ✔ | ✔ | ✔ | ✔ | ✔ | ✔ | ✔ | NA |
| Chi, 2014 [36] | ✔ | ✔ | no | NA | ✔ | ✔ | no | ✔ | NA | ✔ | ✔ | NA | NA | NA | ✔ | no | ✔ | ✔ | no | NA | NA | ✔ | NA | NA | no | ✔ | ✔ | no | ✔ | ✔ | ✔ | ✔ | ✔ | ✔ | ✔ | NA |
| Colombo, 2009 [37] | ✔ | ✔ | ✔ | ✔ | ✔ | ✔ | no | ✔ | NA | no | no | ✔ | no | NA | no | no | ✔ | ✔ | ✔ | ✔ | ✔ | ✔ | ✔ | ✔ | NA | no | ✔ | no | ✔ | ✔ | ✔ | ✔ | ✔ | ✔ | ✔ | NA |
| Costa-Scharplatz, 2015 [38] | ✔ | no | ✔ | ✔ | ✔ | ✔ | no | ✔ | ✔ | NA | ✔ | No | NA | ✔ | no | ✔ | ✔ | ✔ | no | no | NA | ✔ | ✔ | no | NA | no | no | NA | NA | ✔ | no | no | ✔ | ✔ | no | no |
| D’Ausilio, 2015 [39] | ✔ | no | ✔ | ✔ | ✔ | ✔ | no | ✔ | NA | no | ✔ | ✔ | no | NA | no | no | ✔ | ✔ | no | ✔ | no | ✔ | ✔ | ✔ | NA | no | ✔ | no | ✔ | ✔ | ✔ | no | ✔ | ✔ | no | NA |
| D’Souza, 2015 [40] | ✔ | ✔ | no | ✔ | ✔ | ✔ | ✔ | ✔ | NA | ✔ | ✔ | No | NA | NA | no | ✔ | ✔ | ✔ | no | no | no | ✔ | NA | NA | no | no | no | NA | NA | ✔ | no | ✔ | ✔ | ✔ | ✔ | no |
| de Portu, 2010 [41] | ✔ | ✔ | ✔ | ✔ | ✔ | ✔ | ✔ | ✔ | NA | no | ✔ | NA | NA | NA | ✔ | ✔ | ✔ | ✔ | no | no | no | ✔ | NA | NA | no | no | ✔ | no | ✔ | ✔ | ✔ | ✔ | ✔ | ✔ | ✔ | no |
| Feldman, 2003 [42] | ✔ | no | ✔ | ✔ | no | ✔ | ✔ | ✔ | NA | no | ✔ | NA | NA | NA | NA | ✔ | ✔ | ✔ | NA | no | no | ✔ | NA | NA | no | ✔ | ✔ | ✔ | ✔ | ✔ | no | ✔ | ✔ | ✔ | ✔ | ✔ |
| Fernandes, 2012 [43] | ✔ | no | ✔ | ✔ | ✔ | ✔ | no | no | no | no | ✔ | no | no | NA | no | no | ✔ | ✔ | ✔ | ✔ | no | ✔ | ✔ | no | NA | no | ✔ | no | no | ✔ | no | no | ✔ | ✔ | no | no |
| Fernandes, 2012 [44] | ✔ | no | ✔ | ✔ | ✔ | ✔ | no | no | no | no | ✔ | no | no | NA | no | no | ✔ | ✔ | ✔ | ✔ | no | ✔ | ✔ | no | NA | no | ✔ | no | no | ✔ | no | no | ✔ | ✔ | no | no |
| Fernandes, 2012 [45] | ✔ | no | ✔ | ✔ | ✔ | ✔ | no | no | no | no | ✔ | no | no | NA | no | no | ✔ | ✔ | ✔ | ✔ | no | ✔ | ✔ | no | NA | no | ✔ | no | no | ✔ | no | no | ✔ | ✔ | no | no |
| Fernandes, 2012 [46] | ✔ | no | ✔ | ✔ | ✔ | ✔ | no | no | no | no | ✔ | no | no | NA | no | no | ✔ | ✔ | ✔ | ✔ | no | ✔ | ✔ | no | NA | no | ✔ | no | no | ✔ | no | no | ✔ | ✔ | no | no |
| Fernandes, 2012 [47] | ✔ | no | ✔ | ✔ | ✔ | ✔ | no | no | no | no | ✔ | no | no | NA | no | no | ✔ | ✔ | ✔ | ✔ | no | ✔ | ✔ | no | NA | no | ✔ | no | no | ✔ | no | no | ✔ | ✔ | no | no |
| Ferrandiz, 2012 [48] | ✔ | ✔ | ✔ | ✔ | ✔ | ✔ | no | ✔ | NA | ✔ | ✔ | NA | NA | NA | no | ✔ | ✔ | ✔ | no | ✔ | no | ✔ | NA | NA | no | ✔ | ✔ | no | ✔ | ✔ | ✔ | ✔ | ✔ | ✔ | ✔ | no |
| Greiner, 2009 [49] | ✔ | ✔ | ✔ | ✔ | ✔ | ✔ | no | ✔ | NA | no | ✔ | no | NA | NA | ✔ | ✔ | ✔ | ✔ | no | ✔ | no | ✔ | NA | NA | ✔ | no | ✔ | no | ✔ | ✔ | ✔ | ✔ | ✔ | ✔ | ✔ | ✔ |
| Hankin, 2010 [50] | ✔ | ✔ | ✔ | ✔ | ✔ | ✔ | ✔ | ✔ | NA | no | ✔ | no | NA | NA | ✔ | ✔ | ✔ | ✔ | no | no | NA | ✔ | NA | NA | no | no | no | NA | NA | ✔ | no | no | ✔ | ✔ | ✔ | no |
| Heinen-Kammerer, 2007 [51] | ✔ | ✔ | ✔ | no | NA | ✔ | no | ✔ | NA | no | ✔ | ✔ | no | NA | ✔ | no | ✔ | ✔ | no | ✔ | no | ✔ | ✔ | no | NA | no | ✔ | no | ✔ | no | ✔ | ✔ | ✔ | ✔ | ✔ | no |
| Igarashi, 2013 [52] | ✔ | ✔ | ✔ | NA | ✔ | ✔ | no | ✔ | no | ✔ | ✔ | NA | NA | NA | ✔ | ✔ | ✔ | ✔ | no | ✔ | no | ✔ | no | NA | no | ✔ | ✔ | ✔ | ✔ | ✔ | no | ✔ | ✔ | ✔ | ✔ | NA |
| Imafuku, 2017 [53] | ✔ | ✔ | no | ✔ | ✔ | ✔ | no | ✔ | NA | ✔ | ✔ | ✔ | no | NA | no | ✔ | ✔ | ✔ | no | ✔ | no | ✔ | NA | NA | NA | ✔ | no | NA | NA | ✔ | ✔ | ✔ | ✔ | ✔ | ✔ | no |
| Klimes, 2015 [54] | ✔ | ✔ | ✔ | ✔ | ✔ | ✔ | no | ✔ | ✔ | NA | ✔ | ✔ | no | NA | no | ✔ | ✔ | ✔ | ✔ | ✔ | no | ✔ | ✔ | no | NA | ✔ | ✔ | no | no | ✔ | ✔ | ✔ | ✔ | ✔ | ✔ | no |
| Knight, 2012 [55] | ✔ | ✔ | ✔ | ✔ | ✔ | ✔ | no | ✔ | NA | ✔ | ✔ | ✔ | no | ✔ | ✔ | ✔ | ✔ | ✔ | ✔ | ✔ | no | ✔ | ✔ | no | NA | ✔ | ✔ | ✔ | ✔ | ✔ | ✔ | ✔ | ✔ | ✔ | ✔ | ✔ |
| Küster, 2016 [56] | ✔ | ✔ | ✔ | ✔ | ✔ | ✔ | ✔ | ✔ | NA | no | ✔ | NA | NA | ✔ | ✔ | no | ✔ | ✔ | no | ✔ | ✔ | ✔ | ✔ | ✔ | NA | ✔ | ✔ | ✔ | ✔ | ✔ | ✔ | ✔ | ✔ | ✔ | ✔ | ✔ |
| Lee, 2015 [57] | ✔ | no | ✔ | ✔ | ✔ | ✔ | no | ✔ | NA | no | ✔ | ✔ | no | NA | no | no | ✔ | ✔ | no | ✔ | no | ✔ | ✔ | no | NA | no | ✔ | no | ✔ | ✔ | ✔ | ✔ | ✔ | ✔ | no | no |
| Liu, 2012 [58] | ✔ | ✔ | no | no | ✔ | ✔ | no | ✔ | NA | ✔ | ✔ | NA | NA | NA | no | no | ✔ | ✔ | no | ✔ | ✔ | ✔ | NA | NA | no | ✔ | no | NA | NA | no | no | no | ✔ | ✔ | ✔ | no |
| Lloyd, 2009 [59] | ✔ | ✔ | ✔ | ✔ | ✔ | ✔ | no | ✔ | NA | ✔ | ✔ | ✔ | no | NA | no | ✔ | ✔ | ✔ | no | ✔ | ✔ | ✔ | ✔ | ✔ | NA | ✔ | ✔ | no | ✔ | ✔ | ✔ | ✔ | ✔ | ✔ | ✔ | ✔ |
| Martin, 2011 [60] | ✔ | ✔ | no | ✔ | ✔ | ✔ | no | ✔ | ✔ | NA | ✔ | NA | NA | NA | ✔ | ✔ | ✔ | ✔ | no | no | no | ✔ | NA | NA | ✔ | ✔ | ✔ | ✔ | ✔ | ✔ | no | no | ✔ | ✔ | ✔ | no |
| Menter, 2005 [61] | ✔ | ✔ | ✔ | ✔ | ✔ | ✔ | no | ✔ | no | NA | ✔ | NA | NA | NA | ✔ | ✔ | ✔ | ✔ | no | ✔ | no | ✔ | no | NA | no | no | ✔ | no | no | ✔ | no | no | ✔ | ✔ | ✔ | no |
| Mughal, 2015 [62] | ✔ | no | ✔ | ✔ | ✔ | ✔ | no | ✔ | NA | no | ✔ | ✔ | no | NA | no | ✔ | ✔ | ✔ | ✔ | ✔ | no | ✔ | ✔ | no | NA | ✔ | ✔ | no | ✔ | ✔ | ✔ | ✔ | ✔ | ✔ | ✔ | no |
| Nelson, 2006 [63] | ✔ | no | no | ✔ | ✔ | ✔ | no | ✔ | NA | ✔ | ✔ | no | NA | NA | no | no | ✔ | ✔ | no | no | NA | ✔ | NA | NA | no | no | no | NA | NA | ✔ | no | no | ✔ | ✔ | ✔ | ✔ |
| Nelson, 2008 [64] | ✔ | ✔ | ✔ | no | ✔ | ✔ | no | ✔ | NA | ✔ | ✔ | no | NA | NA | no | ✔ | ✔ | ✔ | NA | NA | NA | ✔ | NA | NA | NA | ✔ | ✔ | ✔ | ✔ | ✔ | ✔ | ✔ | ✔ | ✔ | ✔ | NA |
| Pan, 2011 [65] | ✔ | ✔ | ✔ | ✔ | ✔ | ✔ | no | ✔ | ✔ | NA | ✔ | ✔ | no | NA | no | ✔ | ✔ | ✔ | NA | ✔ | ✔ | ✔ | ✔ | no | NA | ✔ | ✔ | ✔ | ✔ | ✔ | ✔ | ✔ | ✔ | ✔ | ✔ | no |
| Pearce, 2006 [66] | ✔ | ✔ | ✔ | ✔ | ✔ | ✔ | no | ✔ | NA | ✔ | ✔ | no | NA | NA | no | ✔ | ✔ | ✔ | no | NA | NA | ✔ | NA | NA | no | no | ✔ | ✔ | ✔ | ✔ | ✔ | ✔ | ✔ | ✔ | ✔ | no |
| Poulin, 2009 [67] | ✔ | ✔ | no | no | ✔ | ✔ | no | ✔ | NA | no | ✔ | no | NA | NA | no | ✔ | ✔ | ✔ | no | NA | NA | ✔ | NA | NA | NA | no | no | NA | NA | ✔ | no | ✔ | ✔ | ✔ | ✔ | NA |
| Puig, 2014 [68] | ✔ | ✔ | no | ✔ | no | ✔ | no | ✔ | no | NA | ✔ | no | NA | NA | no | no | ✔ | ✔ | no | NA | NA | ✔ | NA | NA | no | ✔ | ✔ | no | ✔ | no | ✔ | no | ✔ | ✔ | no | no |
| Puig, 2016 [69] | ✔ | ✔ | no | ✔ | ✔ | ✔ | no | ✔ | ✔ | NA | ✔ | NA | NA | NA | no | no | ✔ | ✔ | no | ✔ | no | ✔ | NA | NA | no | ✔ | ✔ | ✔ | ✔ | no | ✔ | ✔ | ✔ | ✔ | ✔ | no |
| Riveros, 2014 [70] | ✔ | ✔ | ✔ | ✔ | ✔ | ✔ | no | ✔ | NA | ✔ | ✔ | NA | NA | NA | no | ✔ | ✔ | ✔ | ✔ | ✔ | no | ✔ | ✔ | ✔ | NA | ✔ | ✔ | ✔ | ✔ | ✔ | ✔ | ✔ | ✔ | ✔ | ✔ | no |
| Ruano, 2013 [71] | ✔ | ✔ | ✔ | ✔ | ✔ | ✔ | no | ✔ | ✔ | NA | ✔ | NA | NA | ✔ | ✔ | ✔ | ✔ | ✔ | no | no | no | ✔ | NA | NA | no | ✔ | no | NA | NA | ✔ | no | ✔ | ✔ | ✔ | ✔ | no |
| Schmitt-Rau, 2010 [72] | ✔ | ✔ | ✔ | ✔ | ✔ | ✔ | no | ✔ | NA | ✔ | ✔ | NA | NA | NA | ✔ | ✔ | ✔ | ✔ | no | NA | NA | ✔ | NA | NA | no | ✔ | ✔ | no | ✔ | no | ✔ | ✔ | ✔ | ✔ | ✔ | no |
| Sizto, 2009 [73] | ✔ | ✔ | ✔ | ✔ | ✔ | ✔ | no | ✔ | NA | ✔ | ✔ | ✔ | ✔ | ✔ | ✔ | ✔ | ✔ | ✔ | ✔ | ✔ | no | no | no | NA | no | ✔ | ✔ | ✔ | ✔ | ✔ | ✔ | ✔ | ✔ | ✔ | ✔ | no |
| Spandonaro, 2014[74] | ✔ | ✔ | ✔ | ✔ | ✔ | ✔ | no | ✔ | ✔ | NA | ✔ | ✔ | ✔ | NA | no | no | ✔ | ✔ | no | no | no | ✔ | NA | NA | no | ✔ | ✔ | ✔ | ✔ | ✔ | ✔ | ✔ | ✔ | ✔ | ✔ | ✔ |
| Staidle, 2011 [75] | ✔ | ✔ | ✔ | ✔ | ✔ | ✔ | no | ✔ | NA | no | ✔ | NA | NA | NA | ✔ | ✔ | ✔ | ✔ | no | NA | NA | ✔ | NA | NA | no | no | no | NA | NA | ✔ | no | ✔ | ✔ | ✔ | ✔ | ✔ |
| Terranova, 2014 [76] | ✔ | ✔ | ✔ | ✔ | ✔ | ✔ | no | ✔ | no | NA | ✔ | NA | NA | NA | ✔ | ✔ | ✔ | ✔ | no | NA | NA | ✔ | NA | NA | no | ✔ | ✔ | ✔ | ✔ | ✔ | ✔ | ✔ | ✔ | ✔ | ✔ | no |
| Vaatainen, 2015 [77] | ✔ | ✔ | no | ✔ | ✔ | ✔ | no | ✔ | no | NA | ✔ | ✔ | NA | NA | no | no | ✔ | ✔ | no | ✔ | no | ✔ | ✔ | no | NA | ✔ | ✔ | no | no | ✔ | ✔ | ✔ | ✔ | ✔ | no | no |
| Villacorta, 2013 [78] | ✔ | ✔ | ✔ | ✔ | ✔ | ✔ | no | ✔ | ✔ | NA | ✔ | ✔ | ✔ | ✔ | ✔ | ✔ | ✔ | ✔ | ✔ | ✔ | no | ✔ | ✔ | no | ✔ | ✔ | ✔ | ✔ | ✔ | ✔ | ✔ | ✔ | ✔ | ✔ | ✔ | no |
| Wang, 2014 [79] | ✔ | ✔ | ✔ | ✔ | ✔ | ✔ | no | ✔ | NA | ✔ | ✔ | NA | NA | NA | no | no | ✔ | ✔ | no | no | no | ✔ | no | NA | no | ✔ | ✔ | no | ✔ | ✔ | ✔ | no | ✔ | ✔ | ✔ | no |
| Wanke, 2004 [80] | ✔ | no | ✔ | ✔ | ✔ | ✔ | no | no | NA | no | ✔ | NA | NA | NA | no | no | ✔ | ✔ | no | no | no | ✔ | NA | NA | no | no | ✔ | no | no | ✔ | ✔ | no | ✔ | no | no | no |

✔ = Checklist item sufficiently met; no = Checklist item not sufficiently met; NA = not applicable; yr = year of publication.

^a^ The numbers correspond to the checklist items proposed by the Centre for Reviews and Dissemination guidance for undertaking systematic reviews (see S3 Table) [22].
