# Supplementary material for: Biologicals and small molecules in psoriasis: A systematic review of economic evaluations
Source: PLoS One. 2018 Jan 3;13(1):e0189765. doi: 10.1371/journal.pone.0189765 (PMC5751984; doi:10.1371/journal.pone.0189765)
Supplement: S5 Table — (DOCX) [file pone.0189765.s006.docx]

## S5 Table. Study characteristics and methods.

| **Author, Year** | **Country** | **Pers-pective** | **Study type** | **Model design** | **Time horizon** | **Dis-counting^a^** | **Sensitivity analysis** | |
| --- | --- | --- | --- | --- | --- | --- | --- | --- |
|  |  |  |  |  |  |  | **Type** | **Varied parameters** |
| Ahn, 2013 [28] | USA | third party payer | CEA | based on a previous study (Markov model) | 12 weeks, 1 year | NA | one-way, extreme case scenarios | DLQI, PASI 75 response, medication cost, total physician costs, total laboratory costs |
| Alfageme Roldán, 2016 [29] | Spain | societal | CEA | not clear | 12 weeks | NA | no | NA |
| Anis, 2011 [30] | USA | not clear | CUA | Markov model | 1 year | NA | one-way, probabilistic | body weight, hospitalization rate, QALY, dosage of medication, medication cost |
| Armstrong, 2015 [31] | not clear (USA) | not clear | CEA | anchor-based indirect comparison | not clear | NA | no | NA |
| Asche, 2017 [32] | USA | third party payer | CEA | budget impact model | 1 year and 1,000,000 health care plan lives | not clear | one-way | PASI 75 response, medication cost, price discount, length of trial period, number of units after trial period |
| Barbieri, 2015 [33] | Italy | health care system | CUA | Markov model | 5 years | 3% to costs and benefits | one-way, probabilistic | PASI 50, 75, 90 response, dropout rate, utility weights, discount rate, medication cost |
| Blasco, 2009 [34] | Spain | health care system | CEA | Decision tree | 10-24 weeks | NA | extreme case scenarios | PASI 75 response, medication cost |
| Carrascosa, 2015 [35] | Spain | health care system | CUA | Markov model | 20 years | 3% to costs and benefits | one-way, probabilistic | time horizon, discount rate, drug order in the sequence, body weight, cost of infliximab and best supportive care, hospitalization rate |
| Chi, 2014 [36] | USA | not clear | CEA | Decision tree | 6 months | NA | one-way | PASI 75 response, PGA 0/1 |
| Colombo, 2009 [37] | Italy | health care system | CUA | Markov model, based on York model | 10 years | 3,5% to costs and benefits | one-way | cost of etanercept, non-systemic treatment cost, hospitalization cost, efficacy of etanercept, non-systemic treatment efficacy, discount rate |
|  |  |  |  |  |  |  |  |  |
| Costa-Scharplatz, 2015 [38] | Sweden | societal | CEA | not clear | 1, 2, 10 years | 3% to costs and benefits | one-way | not clear |
| D'Ausilio, 2015 [39] | Italy | health care system | CUA | Markov model | 10 years | 3% to costs and benefits | one-way | time horizon, medication cost |
| D'Souza, 2015 [40] | USA | not clear (probably third party payer) | CEA | not clear | 10-24 weeks, 1 year | NA | no | NA |
| de Portu, 2010 [41] | Italy | health care system | CEA | not clear | 24, 50 weeks | NA | one-way, extreme case scenarios | medication cost, PASI 75 response |
| Feldman, 2003 [42] | USA | third party payer | CEA | not clear | 1 year | NA | not clear | PASI 75 response, different dosage regimes |
| Fernandes, 2012 [43] | Argentina | third party payer | CEA, CUA | Decision tree | 96 weeks | 5% to costs and benefits | probabilistic | not clear |
| Fernandes, 2012 [44] | Brazil | (public) third party payer | CEA, CUA | Decision tree | 96 weeks | 5% to costs and benefits | probabilistic | not clear |
| Fernandes, 2012 [45] | Brazil | (private) third party payer | CEA, CUA | Decision tree | 96 weeks | 5% to costs and benefits | probabilistic | not clear |
| Fernandes, 2012 [46] | Colombia | third party payer | CEA, CUA | Decision tree | 96 weeks | 5% to costs and benefits | probabilistic | not clear |
| Fernandes, 2012 [47] | Venezuela | third party payer | CEA, CUA | Decision tree | 96 weeks | 5% to costs and benefits | probabilistic | not clear |
| Ferrandiz, 2012 [48] | Spain | health care system | CEA | Decision tree | 10-24 weeks | NA | extreme case scenarios | body weight, efficacy |
| Greiner, 2009 [49] | Switzer-land | health care system | CEA | Decision tree | 12, 36 weeks | NA | one-way | total treatment costs, efficacy |
| Hankin, 2010 [50] | USA | health care system | CEA | not clear | 1 year | NA | no | NA |
| Heinen-Kammerer, 2007 [51] | Germany | health care system | CUA | Markov model | 10 years | 5% to costs | one-way | cost of basal treatment, medication cost, cost of hospitalization |
| Igarashi, 2013 [52] | Japan | health care system | CEA | York model | 1, 2 years | not clear | one-way | weight, medication cost |
| Imafuku, 2017 [53] | Japan | health care system | CEA | network meta-analysis for outcomes | 12-16 weeks, 1 year | NA | no | NA |
| Klimes, 2015 [54] | Czech Republic | health care system | CUA | Markov model | 10 years | 3% to costs and benefits | probabilistic | efficacy, adverse events, dropout rates, utility weights, costs |
| Knight, 2012 [55] | Sweden | societal | CUA | Markov model | 10 years | 0-5% to costs and benefits | probabilistic | costs (except for medication costs), efficacy |
| Küster, 2016 [56] | Germany | societal | CEA | Markov model | 2 years | 3% to costs | one-way, probabilistic | time horizon, discount rate, efficacy, perspective |
| Lee, 2015 [57] | Canada | health care system | CUA | Markov model | 10 years | 5% to costs and benefits | one-way, probabilistic | medication cost, utility source, drop out rates, perspective, efficacy, other costs (e.g. monitoring) |
| Liu, 2012 [58] | USA | not clear | CEA | Markov model | 12 weeks, 1 year | NA | not clear | NA |
| Lloyd, 2009 [59] | United Kingdom | health care system | CUA | Markov model based on York model | 10 years | 3,5% to costs and benefits | one-way, probabilistic | Patient characteristics (PASI and DLQI at baseline), discount rate, physician cost, hospitalization cost, treatment free interval, retreatment response |
| Martin, 2011 [60] | USA | not clear | CEA | not clear | 16 weeks, 1 year | NA | one-way | time horizon, body weight |
| Menter, 2005 [61] | USA | third party payer | CEA | Decision analytic model | 18 months | not clear | one-way | treatment-free response |
| Mughal, 2015 [62] | United Kingdom (Scotland) | health care system | CUA | Markov model | 10 years | 3,5% to costs and benefits | one-way, probabilistic; scenario with alternative treatment sequence (+ustekinumab, +infliximab) | efficacy, utility weights, hospitalization rate, hospitalization cost, best supportive care cost, discount rates |
| Nelson, 2006 [63] | USA | not clear | CEA | not clear | 12 weeks | NA | no | NA |
| Nelson, 2008 [64] | USA | third party payer | CEA | not clear | 12 weeks | NA | one-way, extreme case scenarios | efficacy, medication cost, physician cost, laboratory cost |
| Pan, 2011 [65] | Canada | health care system | CUA | Markov model | 10 Years | 5% to costs and benefits | one-way, probabilistic | discount rate, time horizon, discontinuation rate, utility values |
| Pearce, 2006 [66] | USA | third party payer | CEA | not clear | 12 weeks | NA | one-way | efficacy |
| Poulin, 2009 [67] | Canada | not clear | CEA | not clear | 12 weeks, 1 year | NA | no | NA |
| Puig, 2014 [68] | Spain | not clear | CEA | not clear | 12-28 weeks | NA | one-way | body weight |
| Puig, 2016 [69] | Spain | not clear | CEA | Decision tree | 1 year | NA | one-way | washout period, dosage variation, body weight, medication cost |
| Riveros, 2014 [70] | Brazil | health care system | CEA | Markov model | 3 years | 5% to costs | one-way, probabilistic | costs and efficacy, time horizon |
| Ruano, 2014 [71] | Spain | societal | CEA | not clear | 1 year | NA | no | NA |
| Schmitt-Rau, 2010 [72] | Germany | third party payer | CEA | not clear | 12 weeks | NA | one-way, extreme case scenarios | efficacy, medication cost |
| Sizto, 2009 [73] | United Kingdom | health care system, societal in sensitivity analysis | CUA | Markov model based on York model | not clear | not clear | one-way, probabilistic | hospitalization, utility valuation, body weight, productivity, dosage of etanercept |
| Spandonaro, 2014 [74] | Italy | health care system | CUA | not clear | 6 months | NA | one-way, probabilistic | hospitalization, physician visits, laboratory and monitoring, utility values |
| Staidle, 2011 [75] | USA | third party payer | CEA | not clear | 1 year | NA | no | NA |
| Terranova, 2014 [76] | Italy | health care system | CEA | not clear | 1 year | NA | one-way, extreme case scenarios | efficacy, cost, body weight, maintenance therapy, dose variation |
| Vaatainen, 2015 [77] | Finland | not clear | CUA | Markov model | 5 years | 3% to costs and benefits | one-way, probabilistic | not clear |
| Villacorta, 2013 [78] | USA | societal | CUA | Markov model | 3 years | 3% to costs and benefits | one-way, probabilistic | time horizon, drug administration, utility valuation, discontinuation, medication cost, mortality |
| Wang, 2014 [79] | Taiwan | health care system | CEA | not clear | 1, 2 years | not clear | extreme case scenarios | efficacy |
| Wanke, 2004 [80] | USA | third party payer | CEA | not clear | 3 months | NA | not clear | medication cost, efficacy rate |

^a^ Discounting was only evaluated if the stated time horizon was more than one year. CEA: cost-effectiveness analysis; CUA: cost-utility analysis; DLQI: Dermatology Life Quality Index; NA: not applicable; PASI: Psoriasis Area and Severity Index; QALY: Quality-Adjusted Life Years.
