# Supplementary material for: Biologicals and small molecules in psoriasis: A systematic review of economic evaluations
Source: PLoS One. 2018 Jan 3;13(1):e0189765. doi: 10.1371/journal.pone.0189765 (PMC5751984; doi:10.1371/journal.pone.0189765)
Supplement: S6 Table — (DOCX) [file pone.0189765.s007.docx]

## S6 Table. Economic results of included studies.

| **Author, Year** | **Comparators** | **Effective-ness measure** | **Utility valu-ation** | **Costs considered** | **Results^a^** | **Results of sensitivity analysis** | **Study conclusions** |
| --- | --- | --- | --- | --- | --- | --- | --- |
| Ahn, 2013 [28] | Adalimumab, alefacept, etanercept, infliximab, ustekinumab in different doses vs. placebo | PASI 75 response and DLQI MID | NA | Medication cost, physician visit, laboratory tests, administration cost | CE for 1 year of treatment:   - Infliximab: 24,066 - 28,444 (PASI 75), 9,727 - 11,348 (DLQI MID) - Adalimumab: 41,691 - 63,112 (PASI 75), 20,055 - 26,871 (DLQI MID) - Ustekinumab: 45,702 - 84,465 (PASI 75), 17,018 - 32,144 (DLQI MID) - Etanercept: 68,272 - 76,906 (PASI 75), 17,931 - 44,796 (DLQI MID) - Alefacept: 131,125 - 200,734 (PASI 75), 14,114 - 155,255 (DLQI MID) | Infliximab stayed the most cost-effective strategy | Infliximab was the most cost-effective option for PASI 75 and DLQI improvement |
| Alfageme Roldán, 2016 [29] | Adalimumab, etanercept, infliximab, ustekinumab, methotrexate, cyclosporine, acitretin, phototherapy vs. placebo and vs. each other | PASI 75 response | NA | Medication cost, first and consecutive physician visit, phototherapy cost, monitoring tests, transportation cost, productivity loss | CE for PASI 75 response (12 weeks):   - Infliximab: 8,077 - Adalimumab: 9,422 - Ustekinumab: 10,634 - Etanercept: 14,338   ICER ustekinumab vs. adalimumab: 16,288 | NA | Methotrexate was the most cost-effective option, phototherapy the least cost-effective. Among biologicals, infliximab was most and etanercept least cost-effective. |
| Anis, 2011 [30] | Adalimumab, alefacept, efalizumab, etanercept, infliximab vs. each other | PASI 50, 75, 90 response | QALY based on EQ-5D and PASI | Medication cost, physician visit, monitoring tests, administration cost, hospitalization, productivity loss | Optimal treatment sequence at ICER-threshold of 50.000 USD:   1. Adalimumab (ICER: 566 USD/QALY vs. Etanercept 25 mg twice weekly) 2. Etanercept 25 mg twice weekly (least costly) 3. Etanercept 50 mg twice weekly (least costly) 4. Infliximab (ICER: 41,353 USD/QALY vs. efalizumab) 5. Efalizumab (least costly) 6. Alefacept (least costly) | In most cases, adalimumab was the most and efalizumab and alefacept were the least cost-effective treatments | Optimal treatment sequence: adalimumab 🡪 etanercept (low-dose) 🡪 etanercept (high-dose) 🡪 infliximab 🡪 efalizumab 🡪 alefacept |
|  |  |  |  |  |  |  |  |
| Armstrong, 2015 [31] | Apremilast vs. methotrexate | PASI 75 response | NA | Medication cost | ICER apremilast vs. methotrexate: 157,309.31 per year | NA | Apremilast exceeds accepted WTP thresholds when compared to methotrexate |
| Asche, 2017 [32] | Etanercept, adalimumab, infliximab, ustekinumab, secukinumab, apremilast, ixekizumab, and calcipotriene/ betamethasone dipropionate foam vs. placebo and above mentioned biologicals vs. calcipotriene/ betamethasone dipropionate foam | PASI 75 response | NA | Medication cost | CE for PASI 75 response (1 year):   - Etanercept: 73,773 - Adalimumab: 92,871 - Infliximab: 34,048 - Ustekinumab: 83,975 - Secukinumab: 113,858 - Apremilast: 47,960 - Ixekizumab: 62,707 - Calcipotriene/betamethasone dipropionate foam: 9,913   ICERs biologicals vs. calcipotriene/betamethasone dipropionate foam:   - Adalimumab: 111,441 - Infliximab: 24,208 - Ustekinumab: 174,841 - Secukinumab: 159,605 - Ixekizumab: 68,829 - Etanercept and apremilast were dominated | Results were sensitive to medication cost, PASI 75 response, trial period and number of units after trial period | Calcipotriene/ betamethasone dipropionate foam was considered a cost-effective strategy. |
| Barbieri, 2015 [33] | Treatment sequence of apremilast 🡪 etanercept 🡪 adalimumab 🡪 ustekinumab 🡪 best supportive care vs. the same sequence without apremilast | PASI 50, 75, 90 response | QALY based on UK NHS utility weights | Medication cost, physician visit, screening and monitoring tests | Starting a treatment sequence with apremilast was a dominant strategy (cost savings: 1,571; 0.01 QALYs gained) | In most cases, the treatment sequence with apremilast was dominant or cost-saving | Apremilast is a cost-saving option without any loss in quality of life |
| Blasco, 2009 [34] | Adalimumab, efalizumab, etanercept, infliximab vs. placebo | PASI 75 response | NA | Medication cost | CE for PASI 75 response (10-24 weeks):   - Adalimumab: 11,983 - Etanercept: 14,013 - 19,138 (depending on dosage and time horizon) - Infliximab: 15,737 - 25,591 (depending on time horizon) - Efalizumab: 21,982 | Adalimumab stayed the most cost-effective drug in best- and worst-case scenarios | Adalimumab is the most cost-effective biological |
| Carrascosa, 2015 [35] | Treatment sequence of apremilast 🡪 adalimumab 🡪 ustekinumab 🡪 etanercept 🡪 best supportive care vs. the same sequence without apremilast | PASI 75 response | QALY based on UK NHS utility weights | Medication cost, administration cost, monitoring tests, hospitalization | Starting a treatment sequence with apremilast was a dominant strategy (cost savings: 9,867; 0.12 QALYs gained) | The treatment sequence with apremilast remained dominant | Starting a treatment sequence with apremilast before other biologicals was a dominant strategy |
| Chi, 2014 [36] | Etanercept, adalimumab, infliximab, and ustekinumab vs. placebo | PASI 75 response, PGA 0/1 | NA | Medication cost | CE for 6 months:   - Adalimumab: 22,040 (PASI 75), 32,006 (PGA 0/1) - Ustekinumab 45 mg: 25,907 (PASI 75), 29,927 (PGA 0/1) - Infliximab: 28,727 (PASI 75), 29,559 (PGA 0/1) - Etanercept: 33,753 (PASI 75), 32,006 (PGA 0/1) - Ustekinumab 90 mg: 48,215 (PASI 75), 55,992 (PGA 0/1) | The order of CE-ratios was not altered by sensitivity analysis | Adalimumab was the most cost-effective option, followed by ustekinumab 45 mg, infliximab, etanercept, and ustekinumab 90 mg |
| Colombo, 2009 [37] | Etanercept vs. non-systemic treatment | PASI 50, 75 response | QALY based on study with TTO | Medication / phototherapy cost, physician visit, monitoring tests, hospitalization + day-hospital admissions | ICER etanercept vs. non-systemic treatment: 46,074 (initial PASI≥10); 35,352 (initial PASI ≥20) | ICERs were robust to sensitivity analysis | Etanercept was a cost-effective option, compared to non-systemic treatment, especially for more severe psoriasis |
| Costa-Scharplatz, 2015 [38] | Secukinumab and ustekinumab vs. placebo | PASI 75, 90 response | NA | Medication cost, physician visit, monitoring tests, administration cost, productivity loss | CE for 2 years of treatment:   - Secukinumab: 26,219 (PASI 75), 34,821 (PASI 90) - Ustekinumab: 62,048 (PASI 75), 100,069 (PASI 90) | Results were robust to sensitivity analysis | Secukinumab was dominant compared to ustekinumab |
|  |  |  |  |  |  |  |  |
| D'Ausilio, 2015 [39] | Secukinumab vs. adalimumab, etanercept, infliximab, ustekinumab and standard of care (cyclosporine and methotrexate) | PASI 75, 90 response | QALY based on Italian health care data | Medication cost, pre-assessment evaluation, physician visit, laboratory/ instrumental tests, hospitalization, management of adverse events | ICER secukinumab:   - dominant vs. infliximab - 1,090/QALY vs. ustekinumab 45 mg - 1,777/QALY vs. ustekinumab 90 mg - 30,243/QALY vs. adalimumab - 35,055/QALY vs. etanercept - 56,380/QALY vs. standard of care | ICERs were robust to sensitivity analysis | ICERs of secukinumab were considered cost-effective according to Italian WTP thresholds |
| D'Souza, 2015 [40] | Etanercept, adalimumab, infliximab, ustekinumab, and other non-biological options vs. placebo | PASI 75 response | NA | Medication cost, physician visit, laboratory tests, administration cost | CE for 1 year:   - Etanercept: 104,206-138,009 - Adalimumab: 43,168-83,344 - Infliximab: 64,057-110,466 - Ustekinumab 45 mg: 68,138-68,955 - Ustekinumab 90 mg: 119,358-136,075 | NA | The most cost-effective option was adalimumab, followed by ustekinumab 45 mg and infliximab, then etanercept and ustekinumab 90 mg |
| de Portu, 2010 [41] | Infliximab vs. etanercept and adalimumab | PASI 75 response, DLQI | NA | Medication cost, administration cost, physician visits | ICER of infliximab   - vs. etanercept stepdown: 3,938 (24 weeks), 5,469 (48 weeks) - vs. etanercept 50 mg biw: dominant (24, 48 weeks) - vs. adalimumab: 22,288 (24 weeks), 31,218 (48 weeks) | ICERs were sensitive to sensitivity analysis | Infliximab was a cost-effective option (acceptable ICERs compared to adalimumab and various dosage regimes of etanercept, dominant compared to high-dose etanercept) |
| Feldman, 2003 [42] | Etanercept, alefacept, infliximab, and other systematic treatments / phototherapy vs. placebo | PASI 75 response | NA | Medication cost, physician visits, laboratory/ instrumental tests, administration cost | CE for 1 year of treatment:   - Etanercept 25 mg twice-weekly: 37,300 - Alefacept: 42,183 - Infliximab 23,378 | CE was comparable for biologicals with infliximab as the most favorable option by trend | Among biological treatments, infliximab showed the most favorable cost-effectiveness |
| Fernandes, 2012 [43] | Etanercept (continuously) and etanercept (intermittently) vs. adalimumab and infliximab, resp. | PASI 75 response | QALY calculation not clear | Medication cost, medical follow-up, management of adverse events | Etanercept (continuously) was dominant compared to infliximab (savings: 17,815);  etanercept (intermittently) was dominant to both infliximab (savings: 26,581) and adalimumab (savings: 8,055) | Etanercept (intermittently) remained the most cost-effective option | Etanercept (intermittently) was a cost-saving strategy with regards to PASI 75 response and QALYs |
| Fernandes, 2012 [44] | Etanercept (intermittently) vs. adalimumab, infliximab, and ustekinumab | PASI 75 response | QALY calculation not clear | Medication cost, medical follow-up, management of adverse events | Etanercept (intermittently) was dominant compared to adalimumab (savings: 7,434), infliximab (savings: 8,488), and ustekinumab (savings: 12,975) | Etanercept (intermittently) remained the most cost-effective option | Etanercept (intermittently) was a cost-saving strategy with regards to PASI 75 response and QALYs |
| Fernandes, 2012 [45] | Etanercept (intermittently) vs. adalimumab and infliximab | PASI 75 response | QALY calculation not clear | Medication cost, medical follow-up, management of adverse events | Etanercept (intermittently) was dominant compared to adalimumab (savings: 21,409) and infliximab (savings: 33,211) | Etanercept (intermittently) remained the most cost-effective option | Etanercept (intermittently) was a cost-saving strategy with regards to PASI 75 response and QALYs |
| Fernandes, 2012 [46] | Etanercept (continuously) and etanercept (intermittently) vs. adalimumab, infliximab, and ustekinumab resp. | PASI 75 response | QALY calculation not clear | Medication cost, medical follow-up, management of adverse events | Etanercept (continuously) was dominant compared to infliximab (savings: 17,137);  etanercept (intermittently) was dominant compared to infliximab, adalimumab and ustekinumab | Etanercept (intermittently) remained the most cost-effective option | Etanercept (intermittently) was a cost-saving strategy with regards to PASI 75 response and QALYs |
| Fernandes, 2012 [47] | Etanercept (continuously) and etanercept (intermittently) vs. adalimumab, infliximab, and ustekinumab resp. | PASI 75 response | QALY calculation not clear | Medication cost, medical follow-up, management of adverse events | Etanercept (continuously or intermittently) was dominant compared to infliximab, adalimumab and ustekinumab | Etanercept (continuously or intermittently) remained the most cost-effective option | Etanercept (continuously or intermittently) was a cost-saving strategy with regards to PASI 75 response and QALYs |
| Ferrandiz, 2012 [48] | Adalimumab, etanercept, infliximab, and ustekinumab vs. placebo | PASI 75 response | NA | Medication cost | - Adalimumab: 11,911 (16 weeks) - Etanercept 25 mg twice/week: 13,542 (12 weeks), 50 mg twice/week: 19,022 (12 weeks) - Infliximab: 15,642 (10 weeks) - Ustekinumab 45 mg: 14,310 (12 weeks), 90 mg: 26,728 (12 weeks) | Results were robust to sensitivity analysis | Adalimumab was the most cost-effective option, followed by etanercept 25 mg twice/week, ustekinumab and infliximab |
| Greiner, 2009 [49] | Infliximab, etanercept, adalimumab, efalizumab, and alefacept vs. placebo | PASI 50, 75, 90 response | NA | Medication cost, administration cost, monitoring tests, adverse events | CE for PASI 75 at 36 weeks:   - Infliximab: 23,075 - Etanercept: 27,386 - Adalimumab: 22,632 - Efalizumab: 25,353 - Alefacept: 37,724 | Results were robust to sensitivity analysis | Adalimumab and infliximab were the most cost-effective options; infliximab was recommended as the first biological therapy from a clinical-economical point of view |
|  |  |  |  |  |  |  |  |
| Hankin, 2010 [50] | Adalimumab, alefacept, efalizumab, etanercept, infliximab, and non-biological treatments vs. placebo | PASI 75 response | NA | Medication cost, administration cost | CE for 1 year:   - Etanercept 25 mg biw: 15,683 - Adalimumab: 19,085 - Infliximab: 21,264 - Etanercept 50 mg biw: 26,020 - Efalizumab: 34,536 - Alefacept: 36,430 | NA | Non-biologic therapies were more cost-effective; among biologicals.  Etanercept low-dose and adalimumab were most, and efalizumab and alefacept least cost-effective. |
| Heinen-Kammerer, 2007 [51] | Etanercept vs. basal treatment | PASI 50, 75 response, DLQI | QALY based on EQ-5D | Medication cost, physician visit, adverse events, hospitalization | ICER for etanercept: 59,069 (baseline PASI and DLQI > 10); 41,626 (baseline PASI and DLQI < 15); 23,572 (baseline PASI and DLQI > 20) | ICER for etanercept remained cost-effective | Etanercept was a cost-effective strategy within accepted WTP thresholds |
| Igarashi, 2013 [52] | Adalimumab, infliximab, and ustekinumab vs. placebo | PASI 50, 75, 90 response | NA | Medication cost | CE for PASI 75:   - Ustekinumab 45 mg: 35,627 (1 year), 30,853 (2 years) - Adalimumab: 40,069 (1year), 38,585 (2 years) - Infliximab: 46,363 (1 year), 38,885 (2 years) | Results were robust to sensitivity analysis | Ustekinumab 45 mg was the most cost-effective option, followed by adalimumab and infliximab |
| Imafuku, 2017 [53] | Adalimumab, secukinumab, ustekinumab, and infliximab vs. placebo | PASI 75, 90 response | NA | Medication cost | CE for PASI 75 response for 12-16 weeks; 1 year:   - Adalimumab: 8,209; 24,626 - Secukinumab: 10,654; 28,411 - Ustekinumab: 15,407; 38,518 - Infliximab: 19,147; 51,059 | NA | Adalimumab and secukinumab were most cost-effective |
| Klimes, 2015 [54] | Secukinumab vs. ustekinumab | PASI 75, 90 response | QALY based on EQ-5D | Medication cost, physician visits, screening and monitoring tests, hospitalization due to exacerbation/ adverse event | Secukinumab was dominant compared to ustekinumab (savings: 206) | In 60% secukinumab remained the dominant strategy | Secukinumab was a dominant strategy compared to ustekinumab when applying PASI 75 as treatment goal |
|  |  |  |  |  |  |  |  |
| Knight, 2012 [55] | Etanercept (intermittently) vs. adalimumab and non-systemic standard of care | PASI 50, 75 response, DLQI | QALY based on EQ-5D and DLQI | Medication cost, administration cost, hospitalization cost, indirect cost (absenteeism and unemployment) | - ICER etanercept vs. non-systemic treatment 10,410 - ICER adalimumab vs. non-systemic treatment: 48,341 - ICER adalimumab vs. etanercept: 173,442 | Etanercept 50 mg was cost-effective in 90%. | Etanercept 50 mg (intermittently) was a more cost-effective strategy compared to adalimumab |
| Küster, 2016 [56] | Adalimumab, ustekinumab, infliximab, etanercept and traditional systemic treatments vs. placebo and vs. each other | PASI 75 response | NA | Medication cost, physician visits, laboratory/ instrumental tests, productivity loss (absenteeism, presenteeism) | CE for 2 years in USD/12 weeks:   - Adalimumab: 9,244 - Ustekinumab: 10,151 (90mg), 10,414 (45mg) - Infliximab: 10,848 - Etanercept: 11,590 (25mg), 12,965 (50mg)   ICER ustekinumab 90mg vs. methotrexate: 18,440; infliximab vs. ustekinumab 90mg: 67,720; adalimumab and etanercept were dominated by ustekinumab | With a 1 year time horizon, adalimumab is a cost-effective option; ustekinumab and infliximab became equally cost-effective | Ustekinumab and infliximab were cost-effective strategies after traditional systemic therapies, adalimumab could be cost-effective depending on the time horizon |
| Lee, 2015 [57] | Secukinumab, adalimumab, etanercept, infliximab, ustekinumab, and standard of care vs. each other | PASI 75, 90 response | QALY based on a Canadian cost-utility study | Medication cost, physician visits, laboratory tests | ICER secukinumab 300 mg vs. standard of care: 72,544, infliximab vs. secukinumab: 863,045; all other biologicals were dominated by standard of care | At a WTP threshold of 90,000 USD secukinumab had the highest probability of being cost-effective | Secukinumab provided increased QALYs at a lower incremental cost compared to etanercept, adalimumab, and ustekinumab. |
| Liu, 2012 [58] | Adalimumab, etanercept, ustekinumab, and infliximab vs. placebo | PASI 75, 90 response | NA | Medication cost, administration cost | CE for PASI 75 response:   - Adalimumab: 10,049 (12 weeks), 36,844 (1 year) - Infliximab: 13,213 (12 weeks), 37,237 (1 year) - Ustekinumab 45 mg: 14,236 (12 weeks) - Etanercept: 22,423 (12 weeks), 59,795 (1 year) - Ustekinumab 90 mg: 26,087 (12 weeks) | NA | Adalimumab was the most cost-effective option (for 12 weeks and 1 year), followed by infliximab, ustekinumab 45 mg, etanercept and ustekinumab 90 mg |
|  |  |  |  |  |  |  |  |
| Lloyd, 2009 [59] | Etanercept (different doses) vs. non-systemic therapy | DLQI | QALY based on EQ-5D and DLQI | Medication cost, physician visit, adverse events, hospitalization | ICER etanercept 50 mg vs. no systemic therapy: 9,183; etanercept 25 mg vs. no systemic therapy: 6,347; etanercept 50mg vs. 25 mg: 17,297 | Results were sensitive to lower re-treatment response. | Etanercept 50mg biw was found cost-effective. |
| Martin, 2011 [60] | Ustekinumab and etanercept vs. placebo | PASI 75 response | NA | Medication cost | - Ustekinumab: 18,449 (16 weeks), 46,122 (1 year) - Etanercept: 20,760 (16 weeks), 47,451 (1year) | In all sensitivity analyses ustekinumab was more cost-effective than etanercept | Ustekinumab was more cost-effective than etanercept |
| Menter, 2005 [61] | Alefacept, efalizumab, and etanercept vs. placebo | PASI 75 response | NA | Medication costs, administration cost, physician visits, laboratory tests, adverse events | CE for 18 months:   - Etanercept: 63,544 - Alefacept: 69,402 - Efalizumab: 78,937 | Results were sensitive to treatment-free response | Cost-effectiveness was similar for etanercept, alefacept and efalizumab |
| Mughal, 2015 [62] | Treatment sequence of apremilast 🡪 adalimumab 🡪 etanercept 🡪 best supportive care vs. the same sequence without apremilast | PASI 50, 75, 90 response | QALY based on UK weights | Medication cost, physician visit, monitoring test, hospitalization, administration cost | Starting a treatment sequence with apremilast is a dominant strategy (cost savings: 4,210, 0.09 QALYs gained) | The sequence with apremilast stayed the dominant strategy in sensitivity analysis and alternative sequences including ustekinumab and infliximab | Starting a treatment sequence with apremilast was a dominant strategy. |
| Nelson, 2006 [63] | Alefacept, efalizumab, etanercept, infliximab, and adalimumab vs. placebo | PASI 75 response, DLQI MID | NA | Medication cost, physician visits, laboratory tests, administration cost | CE for 12 weeks:   - Adalimumab: 8,788 (PASI 75) - Infliximab: 10,139 (PASI 75), 5,210 (DLQI MID) - Etanercept 25 mg biw: 14,352 (PASI 75), 3,414 (DLQI MID) - Efalizumab: 18,892 (PASI 75), 5,478 (DLQI MID) - Alefacept: 77,461 (PASI 75), 28,167 (DLQI MID) | NA | Adalimumab was the most cost-effective option, followed by infliximab, etanercept, efalizumab, and alefacept |
|  |  |  |  |  |  |  |  |
| Nelson, 2008 [64] | Alefacept, efalizumab, etanercept, infliximab, and adalimumab vs. placebo | PASI 75 response, DLQI MID | NA | Medication cost, physician visits, laboratory tests, administration cost | CE for 12 weeks:   - Etanercept: 2,342 (DLQI MID), 19,895 (PASI 75) - Infliximab: 3,652 (DLQI MID); 9,158 (PASI 75) - Adalimumab: 3,655 (DLQI MID); 12,135 (PASI 75) - Efalizumab: 6,831 (DLQI MID), 19,900 (PASI 75) - Alefacept: 28,249 (DLQI MID), 77,685 (PASI 75) | CE was sensitive to changes in medication cost and DLQI efficacy; extreme case scenarios resulted in overlap of biologicals' CE | Adalimumab and infliximab were most cost-effective in terms of PASI 75 response, while etanercept, infliximab, and adalimumab were most cost-effective in terms of DLQI MID |
| Pan, 2011 [65] | Ustekinumab vs. etanercept | PASI 75 response, DLQI | QALY based on EQ-5D and DLQI | Medication cost, physician visits, laboratory test | Ustekinumab was dominant to etanercept (savings: 2,288, 0.0046 QALYs gained) | Ustekinumab remained the dominant strategy | Ustekinumab was the dominant strategy compared to etanercept |
| Pearce, 2006 [66] | Infliximab, etanercept, efalizumab, alefacept and non-biological systemic/ phototherapeutic treatments vs. placebo | PASI 75 response, whole-body clearance | NA | Medication / phototherapy cost, physician visits, laboratory/ instrumental tests, administration cost | CE for 12 weeks:   - Infliximab: 11,139 - Etanercept: 16,981 - Efalizumab: 17,901 - Alefacept: 66,391   All biologicals were dominated by methotrexate | The sequence of biologicals as well as the dominance of methotrexate were robust to sensitivity analysis | Methotrexate was the most cost-effective treatment. Among biologicals infliximab was more cost-effective than etanercept, efalizumab, and alefacept |
| Poulin, 2009 [67] | Adalimumab, alefacept, efalizumab, etanercept, infliximab, and ustekinumab vs. placebo | PASI 75 response | NA | Medication cost | - Adalimumab: 7,325 (12 weeks), 25,638 (1 year) - Ustekinumab: 11,024 (12 weeks), 33,074 (1 year) - Infliximab: 13,284 (12 weeks), 35,425 (1 year) - Etanercept: 15,689 (12 weeks), 41,839 (1 year) - Efalizumab: 15,524 (12 weeks), 67,270 (1 year) - Alefacept: 62,760 (12 weeks), 125,521 (1 year) | NA | Adalimumab was the most cost-effective option, followed by ustekinumab and infliximab |
|  |  |  |  |  |  |  |  |
| Puig, 2014 [68] | Ustekinumab. infliximab, etanercept, and adalimumab vs. placebo | PASI 50, 75 response | NA | Medication cost | CE for PASI 75 at week 24:   - Ustekinumab 45 mg: 15,635 - Adalimumab: 15,903 - Infliximab: 21,880 - Etanercept: 24,241 - Ustekinumab 90 mg: 31,477 | Ustekinumab 45 mg and adalimumab remained the most cost-effective options | Ustekinumab 45 mg and adalimumab were the most cost-effective strategies, compared to infliximab, etanercept, and high-dosage ustekinumab |
| Puig, 2016 [69] | Ustekinumab, infliximab, etanercept, and adalimumab vs. placebo | PASI 50, 75, 90 response | NA | Medication cost, administration cost | CE for PASI 75 at 1 year:   - Ustekinumab: 22,905 - Infliximab: 24,301 - Adalimumab: 25,331 - Etanercept: 30,389 | Ustekinumab remained the most cost-effective option in most analyses | Ustekinumab was considered the most cost-effective option, compared with adalimumab, infliximab, and etanercept. |
| Riveros, 2014 [70] | Adalimumab, infliximab, ustekinumab, and etanercept vs. placebo and vs. each other | PASI 75 response | NA | Medication cost, administration cost, laboratory/ instrumental tests, hospitalization, physician visits, adverse events | CE for 3 years:   - Adalimumab: 73,485 - Ustekinumab: 76,738 - Etanercept: 136,712 - Infliximab: 229,392 - ICER: adalimumab dominates infliximab and etanercept - ICER ustekinumab vs. adalimumab: 102,824 | Results were sensitive to drug cost | Adalimumab was the most cost-effective strategy, closely followed by ustekinumab |
| Ruano, 2014 [71] | Etanercept vs. adalimumab | PASI 75 response | NA | Medication cost, physician visit, monitoring tests, day hospital and hospitalization, transportation, productivity loss | Efficacy and total cost per patient were not significantly different between etanercept (80.0%; 23,896) and adalimumab (85.7%; 24,801) | NA | Etanercept and adalimumab provide similar cost-effectiveness levels |
| Schmitt-Rau, 2010 [72] | Adalimumab, etanercept, infliximab, and ustekinumab vs. placebo | PASI 75 response | NA | Medication cost, physician visits, laboratory/ instrumental costs | CE for 12 weeks:   - Infliximab: 13,560 - Adalimumab: 14,482 - Ustekinumab 45 mg: 16,808 - Etanercept 25mg biw: 21,679 | Extreme case scenarios resulted in overlap of CE, except for etanercept which was less cost-effective | Infliximab was the most cost-effective agent, followed by adalimumab, ustekinumab, and etanercept |
|  |  |  |  |  |  |  |  |
| Sizto, 2009 [73] | Efalizumab, etanercept, infliximab, adalimumab, and other non-biological systemic treatments vs. supportive care and biologicals vs. each other | PASI 50, 75, 90 response | QALY based on EQ-5D and PASI | Medication cost, administration cost, monitoring cost, hospitalization; productivity loss in sensitivity analysis | ICER vs. supportive care:   - Adalimumab: 45,109 - Etanercept: 55,653 - Efalizumab: 59,009 - Infliximab: 62,767   ICER vs. each other: adalimumab dominates etanercept and efalizumab; ICER infliximab vs. adalimumab: 218,478 | Results were sensitive, especially to hospitalization rate and productivity loss. | Among biological treatments, adalimumab was most cost-effective |
| Spandonaro, 2014 [74] | Etanercept, adalimumab, and infliximab vs. placebo | PASI response | QALY based on EQ-5D | Medication cost, hospitalization, day hospital, physician visits, laboratory and monitoring costs | CE for 6 months:   - Etanercept: 35,251 - Adalimumab: 39,952 - Infliximab: 73,021 | Results were robust, staying under accepted WTP thresholds in 96.2% | Biological treatment in general was cost-effective, among those etanercept was the most effective, followed by adalimumab and infliximab |
| Staidle, 2011 [75] | Etanercept, infliximab, adalimumab, alefacept, and ustekinumab vs. placebo | PASI 75 response, DLQI MID | NA | Medication cost, physician visits, laboratory and monitoring costs | CE for 1 year:   - Infliximab: approx. 22,700 (PASI 75), approx. 10,300 (DLQI MID) - Ustekinumab: approx. 35,000 (PASI 75), approx. 15,500 (DLQI MID) - Adalimumab: approx. 39,000 (PASI 75), approx. 15,500 (DLQI MID) - Etanercept 50 mg biw: approx. 94,000 (PASI 75), approx. 38,200 (DLQI MID) - Alefacept: 129,043 (PASI 75), 61,589 (DLQI MID) | NA | Among biological treatments, infliximab was most cost-effective, followed by ustekinumab and adalimumab |
| Terranova, 2014 [76] | Ustekinumab, infliximab, adalimumab, and etanercept vs. placebo and vs. each other | PASI 75 response | NA | Medication cost, monitoring cost, administration, physician visits | CE for 1 year:   - Ustekinumab: 29,139 (45mg), 28,293 (90 mg) - Adalimumab: 32,018 - Infliximab: 32,213 - Etanercept: 38,943   ICER ustekinumab 45mg vs. adalimumab: 14,476; ustekinumab was dominant compared to etanercept 50 mg and infliximab | Results were robust to sensitivity analysis | Ustekinumab was the most cost-effective option |
| Vaatainen, 2015 [77] | Treatment sequence of ustekinumab 🡪 adalimumab 🡪 etanercept 🡪 infliximab 🡪 maintenance vs. the same sequence without ustekinumab | PASI response | QALY based on EQ-5D and DLQI / PASI | Medication cost, administration cost, laboratory tests, physician visits, adverse events, direct costs to patient | Starting a treatment sequence with ustekinumab was the dominant strategy (cost savings: 2,776; 0.07 QALYs gained) | Dominance of the ustekinumab sequence was considered robust | Starting a treatment sequence with ustekinumab was considered cost-effective |
| Villacorta, 2013 [78] | Ustekinumab vs. etanercept | PASI response | QALY based on PASI (TTO) | Medication cost, physician visit, administration cost, adverse events, productivity loss (traveling, waiting time) | ICER vs. etanercept 50 mg:   - Ustekinumab 45 mg was dominant (savings: 4,037; 0.04 QALYs gained) - Ustekinumab 90 mg: 395,933 | Most sensitivity analyses resulted in dominance of ustekinumab 45 mg over etanercept 50 mg | Ustekinumab 45 mg was the most cost-effective strategy compared to etanercept 50 mg |
| Wang, 2014 [79] | Etanercept, adalimumab, and ustekinumab vs. placebo | PASI 75 response | NA | Medication cost | CE for 1 year:   - Adalimumab: 23,921 - Ustekinumab: 26,562 - Etanercept: 40,061 | Extreme case scenarios showed overlapping CE for adalimumab and ustekinumab | Adalimumab and ustekinumab were both cost-effective options, etanercept was the least cost-effective option |
| Wanke, 2004 [80] | Etanercept vs. alefacept | PASI 50, 75 response | NA | Medication cost, physician visits, laboratory tests, drug administration, adverse events | CE for PASI 75 at 3 months:   - Etanercept: 14,290 - 18,324 - Alefacept: 83,365 - 98,641   ICER: etanercept was dominant compared to alefacept | Results were sensitive to drug cost and efficacy rate | Etanercept was more cost-effective than alefacept |

^a^ Results were updated to 2015 USD, only partial study results are displayed. biw: biweekly; CE: cost-effectiveness; DLQI: Dermatology Life Quality Index; ICER: Incremental cost-effectiveness ratio; MID: minimal important difference; NA: not applicable; PASI: Psoriasis Area and Severity Index; QALY: Quality-Adjusted Life Years; resp.: respectively; TTO: time trade-off ; WTP: willingness-to-pay.
