# Supplementary material for: Biologicals and small molecules in psoriasis: A systematic review of economic evaluations
Source: PLoS One. 2018 Jan 3;13(1):e0189765. doi: 10.1371/journal.pone.0189765 (PMC5751984; doi:10.1371/journal.pone.0189765)
Supplement: S7 Table — (DOCX) [file pone.0189765.s008.docx]

## S7 Table. Comparators to favored biologicals according to study conclusions.

| **Comparator to treatment^b^ 🡪**  **Preferred biological^a^ 🡻** | **Adalimumab**  **n (%)** | **Alefacept**  **n (%)** | **Apremilast**  **n (%)** | **Efalizumab**  **n (%)** | **Etanercept**  **n (%)** | **Infliximab**  **n (%)** | **Ixekizumab**  **n (%)** | **Secukinumab**  **n (%)** | **Ustekinumab**  **n (%)** | **Overall number of studies^c^** |
| --- | --- | --- | --- | --- | --- | --- | --- | --- | --- | --- |
| **Adalimumab** | NA | 6 (37.5) | 0 (0) | 8 (50) | 15 (93.8) | 15 (93.8) | 0 (0) | 1 (6.3) | 9 (56.3) | 16 |
| **Apremilast** | 3 (100) | 0 (0) | NA | 0 (0) | 3 (100) | 0 (0) | 0 (0) | 0 (0) | 2 (66.7) | 3 |
| **Etanercept** | 8 (44.4) | 2 (11.1) | 0 | 1 (5.6) | NA | 7 (38.9) | 0 (0) | 0 (0) | 3 (16.7) | 18 |
| **Infliximab** | 9 (81.8) | 5 (45.5) | 1 (9.1) | 3 (27.3) | 11 (100) | NA | 1 (9.1) | 1 (9.1) | 6 (54.5) | 11 |
| **Secukinumab** | 2 (50) | 0 (0) | 0 (0) | 0 (0) | 2 (50) | 2 (50) | 0 (0) | NA | 2 (50) | 4 |
| **Ustekinumab** | 7 (70) | 0 (0) | 0 (0) | 0 (0) | 9 (90) | 6 (60) | 0 (0) | 0 (0) | NA | 10 |

^a^ Preferred biological according to study conclusions. ^b^ Drug used as comparator to the preferred biological. ^c^ Overall number of studies favoring each medication (see also Table 5). Alefacept, efalizumab, and ixekizumab were not preferred in any study conclusion. Etanercept was frequently included as comparator in studies that favored other biologicals. Recently approved medications, i.e., apremilast, ixekizumab, and secukinumab, were rarely used as comparators. n: number of studies; %: percentage of the total number of studies favoring the preferred biological. For example, alefacept was included as comparator in 6 of 16 studies (37.5%) that favored adalimumab in the study conclusions. NA: not applicable.
