# Supplementary material for: Biologicals and small molecules in psoriasis: A systematic review of economic evaluations
Source: PLoS One. 2018 Jan 3;13(1):e0189765. doi: 10.1371/journal.pone.0189765 (PMC5751984; doi:10.1371/journal.pone.0189765)
Supplement: S8 Table — (DOCX) [file pone.0189765.s009.docx]

## S8 Table. Funding information.

| **Author, Year** | **Funder^a^** | **Funder’s medical product for psoriasis** | **Consistency of funder’s interest and study result** |
| --- | --- | --- | --- |
| Ahn, 2013 [28] | None | None | NA |
| Alfageme Roldán, 2016 [29] | None | None | NA |
| Anis, 2011 [30] | Abbott Laboratories | Abbott sold Humira® (adalimumab) until foundation of AbbVie | Yes, adalimumab was recommended as first treatment in the sequence |
| Armstrong, 2015 [31] | AbbVie | AbbVie sells Humira® (adalimumab) | Not clear, apremilast (biological of the competitor Celgene) was considered as not cost-effective. |
| Asche, 2017 [32] | LEO Pharma | LEO Pharma sells Enstilar Foam® (calcipotriene/betamethasone dipropionate foam) | Yes, calcipotriene/betamethasone dipropionate foam was considered a cost-effective. |
| Barbieri, 2015 [33] | Celgene | Celgene sells Otezla® (apremilast) | Yes, apremilast was recommended as a cost-saving option |
| Blasco, 2009 [34] | Abbott Laboratories | Abbott sold Humira® (adalimumab) | Yes, adalimumab was more cost-effective than etanercept, infliximab, and efalizumab. |
| Carrascosa, 2015 [35] | Celgene | Celgene sells Otezla® (apremilast) | Yes, apremilast was recommended as a dominant option |
| Chi, 2014 [36] | None | None | NA |
| Colombo, 2009 [37] | Wyeth Lederle | Wyeth Lederle (now Pfizer) sells Enbrel® (etanercept) | Yes, ICER for etanercept therapy were below accepted thresholds |
| Costa-Scharplatz, 2015 [38] | 3 of 4 authors worked for Novartis | Novartis sells Cosentyx® (secukinumab) | Yes, secukinumab was considered the dominant strategy |
| D'Ausilio, 2015 [39] | Novartis | Novartis sells Cosentyx® (secukinumab) | Yes, secukinumab was considered cost-effective |
| D'Souza, 2015 [40] | None | None | NA |
| de Portu, 2010 [41] | None | None | NA |
| Feldman, 2003 [42] | None | None | NA |
| Fernandes, 2012 [43] | 1 of 5 authors worked for Pfizer (funding not explicitly stated) | Pfizer sells Enbrel® (etanercept) | Yes, etanercept was considered the most cost-effective option |
| Fernandes, 2012 [44] | 5 of 9 authors worked for Pfizer (funding not explicitly stated) | Pfizer sells Enbrel® (etanercept) | Yes, etanercept was considered the most cost-effective option |
| Fernandes, 2012 [45] | 5 of 9 authors worked for Pfizer (funding not explicitly stated) | Pfizer sells Enbrel® (etanercept) | Yes, etanercept was considered the most cost-effective option |
| Fernandes, 2012 [46] | 3 of 7 authors worked for Pfizer (funding not explicitly stated) | Pfizer sells Enbrel® (etanercept) | Yes, etanercept was considered the most cost-effective option |
| Fernandes, 2012 [47] | 2 of 6 authors worked for Pfizer (funding not explicitly stated) | Pfizer sells Enbrel® (etanercept) | Yes, etanercept was considered the most cost-effective option |
| Ferrandiz, 2012 [48] | Abbott Laboratories | Abbott sold Humira® (adalimumab) | Yes, adalimumab was considered the most cost-effective option |
| Greiner, 2009 [49] | Essex Chemie AG | Essex (subcompany of Schering-Plough/MSD) sells Remicade® (infliximab) | Yes, infliximab was considered the best biological treatment option to start with |
| Hankin, 2010 [50] | Stiefel Laboratories | Stiefel Laboratories sells Soriatane® (acitretin) | Yes, biologicals were considered less cost-effective than traditional systemic medication (such as acitretin) |
| Heinen-Kammerer, 2007 [51] | Wyeth Pharma | Wyeth Pharmaceuticals (now Pfizer) sells Enbrel® (etanercept) | Yes, etanercept was considered a cost-effective treatment option |
| Igarashi, 2013 [52] | Janssen Pharmaceutical K.K. | Janssen-Cilag sells Stelara® (ustekinumab) | Yes, ustekinumab was considered the most cost-effective option |
| Imafuku, 2017 [53] | AbbVie | AbbVie sells Humira® (adalimumab) | Yes, adalimumab was considered to be cost-effective |
| Klimes, 2015 [54] | 7 of 8 authors worked for Novartis (funding not explicitly stated) | Novartis sells Cosentyx® (secukinumab) | Yes, secukinumab was considered the dominant strategy |
| Knight, 2012 [55] | 2 of 6 authors worked for Pfizer (funding not explicitly stated) | Pfizer sells Enbrel® (etanercept) | Yes, etanercept 50 mg was considered cost-effective compared to adalimumab and non-systemic treatment |
| Küster, 2016 [56] | MSD | MSD sells Remicade® (infliximab) | Yes, infliximab was considered a cost-effective strategy |
| Lee, 2015 [57] | 2 of 5 authors worked for Novartis (funding not explicitly stated) | Novartis sells Cosentyx® (secukinumab) | Yes, secukinumab was considered a cost-effective strategy |
| Liu, 2012 [58] | Abbott Laboratories | Abbott sold Humira® (adalimumab) | Yes, adalimumab was considered the most cost-effective option |
| Lloyd, 2009 [59] | 3 of 5 authors worked for Wyeth Pharmaceuticals | Wyeth Pharmaceuticals (now Pfizer) sells Enbrel® (etanercept) | Yes, etanercept 50 mg was considered cost-effective |
| Martin, 2011 [60] | Centocor R&D, Johnson & Johnson | Centocor R&D is a subsidiary of Johnson & Johnson, Johnson & Johnson's subsidiary Janssen-Cilag sells Stelara® (ustekinumab) | Yes, ustekinumab was considered the most cost-effective option |
| Menter, 2005 [61] | None | None | NA |
| Mughal, 2015 [62] | Celgene | Celgene sells Otezla® (apremilast) | Yes, apremilast was considered a dominant strategy at the beginning of a treatment sequence |
|  |  |  |  |
| Nelson, 2006 [63] | Galderma Laboratories | No, Galderma sells topical treatments for psoriasis. | NA |
| Nelson, 2008 [64] | Galderma Laboratories | No, Galderma sells topical treatments for psoriasis | NA |
| Pan, 2011 [65] | Janssen | Janssen-Cilag sells Stelara® (ustekinumab) | Yes, ustekinumab was considered the most cost-effective option |
| Pearce, 2006 [66] | Galderma Laboratories | No, Galderma sells topical treatments for psoriasis | NA |
| Poulin, 2009 [67] | Abbott Laboratories | Abbott sold Humira® (adalimumab) | Yes, adalimumab was considered the most cost-effective option |
| Puig, 2014 [68] | None | None | NA |
| Puig, 2016 [69] | Janssen-Cilag | Janssen-Cilag sells Stelara® (ustekinumab) | Yes, ustekinumab was considered the most cost-effective option |
| Riveros, 2014 [70] | None | None | NA |
| Ruano, 2014 [71] | None | None | NA |
| Schmitt-Rau, 2010 [72] | None | None | NA |
| Sizto, 2009 [73] | Abbott Laboratories | Abbott sold Humira® (adalimumab) | Yes, adalimumab was considered the most cost-effective option |
| Spandonaro, 2014 [74] | Pfizer | Pfizer sells Enbrel® (etanercept) | Yes, etanercept was considered a cost-effective treatment option |
| Staidle, 2011 [75] | Galderma Laboratories | No, Galderma sells topical treatments for psoriasis | NA |
| Terranova, 2014 [76] | None | None | NA |
| Vaatainen, 2015 [77] | Janssen-Cilag | Janssen-Cilag sells Stelara® (ustekinumab) | Yes, starting a treatment sequence with ustekinumab was considered to be cost-effective |
| Villacorta, 2013 [78] | 1 of 3 authors worked part-time for Johnson & Johnson | Janssen-Cilag (subsidiary of Johnson & Johnson) sells Stelara® (ustekinumab) | Yes, ustekinumab was the most cost-effective option |
| Wang, 2014 [79] | None | None | NA |
| Wanke, 2004 [80] | Amgen | Amgen sells Enbrel® (etanercept) in the USA and Canada | Yes, etanercept was considered more cost-effective than alefacept |

^a^ Funders of studies and/or authors who were employed by pharmaceutical companies are mentioned in this table. Honoraria from pharmaceutical companies provided to other authors were not considered if they were payed for other purposes than for the study cited. A large number of authors stated in the conflict of interest declaration that they had received honoraria from various competing companies. NA: not applicable.
